# Tumor inherent interferon regulators as biomarkers of long-term chemotherapeutic response in TNBC

**DOI:** 10.1038/s41698-019-0093-2

**Published:** 2019-08-29

**Authors:** Natasha K. Brockwell, Jai Rautela, Katie L. Owen, Linden J. Gearing, Siddhartha Deb, Kate Harvey, Alex Spurling, Damien Zanker, Chia-Ling Chan, Helen E. Cumming, Niantao Deng, Jasmine M. Zakhour, Hendrika M. Duivenvoorden, Tina Robinson, Marion Harris, Michelle White, Jane Fox, Corinne Ooi, Beena Kumar, Jacqui Thomson, Nicole Potasz, Alex Swarbrick, Paul J. Hertzog, Tim J. Molloy, Sandra O’ Toole, Vinod Ganju, Belinda S. Parker

**Affiliations:** 1 Department of Biochemistry and Genetics, La Trobe Institute for Molecular Science, La Trobe University, Melbourne, VIC Australia; 2 Sir Peter MacCallum Department of Oncology, University of Melbourne, Parkville, Australia; 3 Cancer Immunology and Therapeutics Programs, Peter MacCallum Cancer Centre, Melbourne, Australia; 4 The Walter and Eliza Hall Institute of Medical Research, Parkville, VIC Australia; 5 Department of Medical Biology, University of Melbourne, Melbourne, VIC Australia; 6 Centre for Innate Immunity and Infectious Diseases, Hudson Institute of Medical Research, Clayton, VIC Australia; 7 Department of Molecular and Translational Science, Monash University, Clayton, VIC Australia; 8 Anatpath, Gardenvale, VIC Australia; 9 Cancer Research Division, The Kinghorn Cancer Centre/Garvan Institute of Medical Research, Sydney, NSW Australia; 10 Monash Health, Clayton, VIC Australia; 11 Monash Health School of Clinical Sciences, Monash University, Clayton, VIC Australia; 12 Penninsula Health, Frankston, VIC Australia; 13 St Vincent’s Clinical School, Faculty of Medicine, University of New South Wales, Sydney, NSW Australia; 14 St Vincent’s Centre for Applied Medical Research, Darlinghurst, NSW Australia; 15 Department of Tissue Pathology and Diagnostic Oncology, Royal Prince Alfred Hospital, Sydney, NSW Australia; 16 Sydney Medical School, University of Sydney, Sydney, NSW Australia; 17 Australian Clinical Labs, Bella Vista, NSW Australia

**Keywords:** Tumour biomarkers, Breast cancer, Prognostic markers

## Abstract

Patients diagnosed with triple negative breast cancer (TNBC) have an increased risk of rapid metastasis compared to other subtypes. Predicting long-term survival post-chemotherapy in patients with TNBC is difficult, yet enhanced infiltration of tumor infiltrating lymphocytes (TILs) has been associated with therapeutic response and reduced risk of metastatic relapse. Immune biomarkers that predict the immune state of a tumor and risk of metastatic relapse pre- or mid-neoadjuvant chemotherapy are urgently needed to allow earlier implementation of alternate therapies that may reduce TNBC patient mortality. Utilizing a neoadjuvant chemotherapy trial where TNBC patients had sequential biopsies taken, we demonstrate that measurement of T-cell subsets and effector function, specifically CD45RO expression, throughout chemotherapy predicts risk of metastatic relapse. Furthermore, we identified the tumor inherent interferon regulatory factor IRF9 as a marker of active intratumoral type I and II interferon (IFN) signaling and reduced risk of distant relapse. Functional implications of tumor intrinsic IFN signaling were demonstrated using an immunocompetent mouse model of TNBC, where enhanced type I IFN signaling increased anti-tumor immunity and metastasis-free survival post-chemotherapy. Using two independent adjuvant cohorts we were able to validate loss of IRF9 as a poor prognostic biomarker pre-chemotherapy. Thus, IRF9 expression may offer early insight into TNBC patient prognosis and tumor heat, allowing for identification of patients that are unlikely to respond to chemotherapy alone and could benefit from further immune-based therapeutic intervention.

## Introduction

Triple negative breast cancer (TNBC) accounts for 15–20% of all breast cancers and is considered an aggressive subtype due to the greater risk of metastasis within the first few years of diagnosis compared to other subtypes.^1–3^ With a lack of targetable receptors, TNBC patients remain heavily reliant on combination chemotherapy (commonly incorporating taxanes and anthracyclines^4^) and there are limited alternatives for those who fail to respond or progress to metastatic disease. In the neoadjuvant setting, 15–35% of patients have a complete pathological response to chemotherapy (cPR) and this is closely associated with a favorable prognosis.^5–8^ However, predicting response to chemotherapy pre-treatment and those patients at most risk of relapse after a partial or lack of response to chemotherapy is difficult and is an essential area of research for individualized treatment strategies in this subtype. This is also important in adjuvant setting where cPR cannot be measured.

As with a number of other solid malignancies, enhanced accumulation of tumor infiltrating lymphocytes (TILs) has been associated with favorable outcome and response to chemotherapy in TNBC.^9–11^ Assessment of the specific nature of the tumor immune infiltrate has been demonstrated superior for predicting disease progression. CD8^+^ T lymphocytes are key players in anti-tumor immunity and their intratumoral accumulation has been linked to favorable outcomes in a number of cancers, including breast, where their high proportions correlate with enhanced survival and response to chemotherapy.^12,13^ Further interrogation of these populations has revealed that antigen experienced T cells (CD45RO^+^ CD8^+^) predict favorable outcomes in non-small cell lung cancer and colorectal cancer.^14–16^ New advances in single-cell immune profiling have also supported a link between particular CD8^+^ T cell subsets and reduced risk of disease progression, whereby an enhanced tissue resident memory T-cell signature (T_RM_) was associated with improved patient survival in TNBC.^17^ In support of this, treatment success in melanoma and other cancers with inherently high mutational load has been attributed to a “T cell inflamed” or “hot” tumor microenvironment (TME), associated with enhanced infiltration of CD8^+^ T cells along with B cells, macrophages, FOXP3^+^ cells, and type I and II IFN signatures.^18,19^

The link between type I IFN signatures and the T-cell inflamed tumor is not surprising as IFNs are well-known immunoactivating cytokines that can influence immune reactivity and response to therapies, including chemotherapy.^20–22^ Type I IFNs induce a multitude of interferon regulated genes (IRGs) that can impact accumulation, activation, and function of immune cells, and also directly act on tumor cells via anti-proliferative and pro-apoptotic functions.^21,23–25^ Loss of host IFN signaling, global or cell specific, has been linked to cancer initiation, progression, and metastasis in a number of solid malignancies.^26–29^ Our previous studies in breast cancer discovered tumor cell intrinsic interferon signaling as a critical mediator of the anti-tumor immune response,^29^ the loss of which promotes immune escape and bone metastasis.^26^ Accumulating evidence also supports tumor inherent IFN signals as key readouts of therapeutic response. In mouse models of TNBC, anthracycline-based chemotherapeutic response has been demonstrated reliant on tumor inherent type I IFN signaling and induction.^30,31^ Further, the presence of a type I IFN metagene predicted complete response to neoadjuvant anthracycline-based therapy in patients with TNBC.^30^ This link between IFNs and therapeutic response has also been explored in the immunotherapy context in melanoma, where the presence of a type I IFN signature was associated with response to anti-PD-1,^32^ a checkpoint inhibitor being met with limited success as a single agent in TNBC. This is supported by our studies in murine models of TNBC whereby response to anti-PD-1 relied on combination therapy with type I IFN inducers, promoting a long-term tumor-specific T-cell response.^28,29,33^ Together, these studies highlight type I IFNs as crucial regulators of the immunoreactivity of the TME and potential biomarkers of therapeutic response.

While immune infiltrates and type I IFN signatures have both been associated with improved therapy responses, the discovery of prognostic biomarkers encompassing them has been limited. TME immune characterization along with the interrogation of IFN signaling and the association with prognosis and therapeutic response in TNBC could offer important insight into their potential utility as biomarkers. This study investigates the role of tumor inherent IFN signaling in tumor progression and metastatic risk post-chemotherapy in clinical samples and mouse models of TNBC. Using a clinical sequential biopsy cohort, we characterize the TME throughout chemotherapeutic administration to profile immune markers of therapeutic benefit and risk of metastatic relapse. We highlight the association of tumor resident T cells along with tumor cell intrinsic type I IFN signals with a favorable prognosis in TNBC, and identify interferon regulatory factor 9 (IRF9) as a candidate prognostic biomarker in this subtype. Reduced IRF9 expression predicted poor outcome in TNBC despite chemotherapy.

## Results

### Presence of tumor infiltrating memory cells correlates with chemotherapeutic response in TNBC pre-therapy

**Fig. 1:**
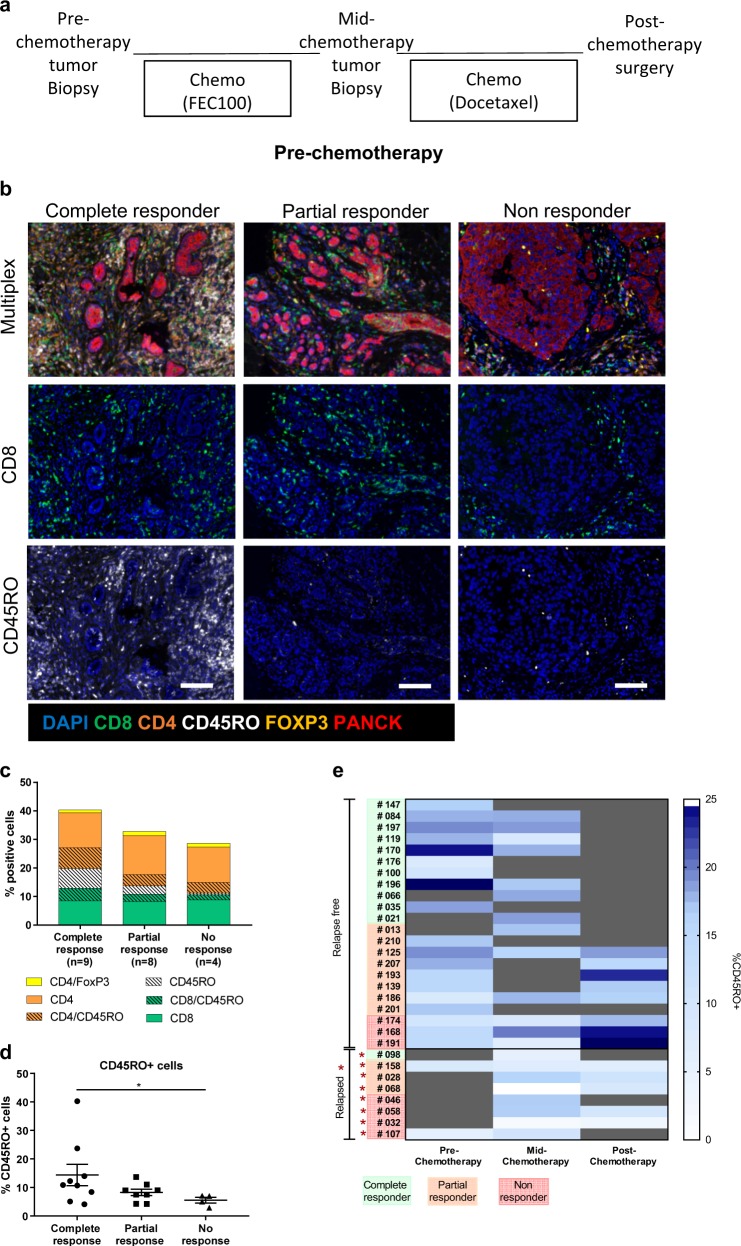
Profiling the immune landscape throughout chemotherapy. **a** Outline of the SETUP trial. **b** Representative images of complete, partial and non-responder TNBC primary tumors pre-chemotherapy. Sections measuring 3 μm were co-stained for expression of CD8 (green), CD4 (orange), CD45RO (white), FOXP3 (yellow), and PanCK (red) followed by counterstain using DAPI to visualize cell nuclei. Images are shown as multiplexed fluorescent images or single-fluorescent images (CD8, CD45RO). Scale bars represent 100 μm. **c** Bar graph of the mean proportion of immune populations determined by inForm software in complete, partial and non-responder TNBC primary tumors pre-chemotherapy. **d** Percentage of CD45RO^+^ cells in the stroma compared in complete, partial and non-responder TNBC primary tumors pre-chemotherapy. **e** Heat map representing the percentage of CD45RO^+^ cells in the stroma of TNBC primary tumors throughout chemotherapy. Gray shading indicates no sample for evaluation. Error bars represent SEM. *p < 0.05 using Mann–Whitney *U* *test*

We first evaluated the association of immune infiltrate with chemotherapeutic response and long-term survival via retrospective analysis of the SETUP (Sequential Evaluation of Tumors Undergoing Preoperative chemotherapy) clinical trial where patients had biopsies taken pre- and mid-chemotherapy followed by total excision post-chemotherapy (Fig. 1a; Supplementary Table S1). We utilized the TNBC patient arm comprising 35 patients, number of patients with evaluable FFPE tissue at each time point is highlighted in the REMARK diagram (Supplementary Fig. 1). Trial outcome was based on both tumor response and distant relapse-free survival. For assessment of tumor response, patients were classed as having a pathological complete response (no detectable tumor at time of surgery), partial response (decrease in tumor size/grade), or no response (no change in tumor size/grade or progression). Due to matched sample disparity (Supplementary Fig. 1) each biopsy time point was analyzed independently to predict the link between immune status and long-term outcomes at various stages of chemotherapeutic administration. All samples with detectable tumor were subject to multiplex immunohistochemistry (IHC) using the OPAL method, prior to whole slide VECTRA scanning and representative images taken per tissue section (Supplementary Fig. 2a). Representative images were spectrally unmixed (Supplementary Fig. 2b, c), segmented into tumor (red), stroma (green) (Supplementary Fig. 2d), and single cells (Supplementary Fig. 2e). Multiplex (Supplementary Fig. 2c) or single-fluorescent view images illustrate the proportion of tumor cells and immune cells stained (Supplementary Fig. 2f–j).

TIL characterization revealed differences in the overall immune landscape between complete, partial and non-responders, presented in representative images (Fig. 1b) and analysis of immune populations present pre-chemotherapy (Fig. 1c; Supplementary Fig. 3a). Immune characterization revealed that pre-chemotherapy measurement of specific infiltrates was more closely associated with chemotherapeutic response than TIL score alone in this small cohort (Supplementary Fig. 3b, c). Most notably, the proportion of CD45RO^+^ cells increased in tumors of complete responders compared to non-responders (Fig. 1d; Supplementary Fig. 3d) pre-chemotherapy. CD45RO is an immune memory marker, whereby expression in T cells suggests induction of a T-cell response at the site of a tumor. Analysis of other immune populations revealed no difference in abundance between complete, partial, and non-responders pre-chemotherapy (Supplementary Fig. 3e–i). Comparison of CD45RO expression in tumors based on relapse suggested dampened infiltrate in relapsing tumors as indicated by the cool colors in the heat map, this was also seen at mid-chemotherapy and post-chemotherapy (Fig. 1e). This pattern was also apparent when comparing the proportion of double positive CD8^+^ CD45RO^+^ cells throughout chemotherapy (Supplementary Fig. 3j).

The relationship between specific immune infiltrates and chemotherapeutic response was not evident mid-chemotherapy (Supplementary Fig. 4a–d) and post-chemotherapy in this small cohort (Supplementary Fig. 4e–h). Post-chemotherapy only partial and non-responders were analyzed due to absence of tumor in complete responders. However, when patients were categorized based on relapse, overall TIL characterization suggested that patients who remained relapse-free had more T-cell infiltrates than relapsed patients mid-chemotherapy and post-chemotherapy (Supplementary Fig. 4i, j). These data suggest that immune infiltrate may predict distant relapse independent of chemotherapeutic pathological response.

### Immune landscape predicts distant relapse during and post-chemotherapy

**Fig. 2:**
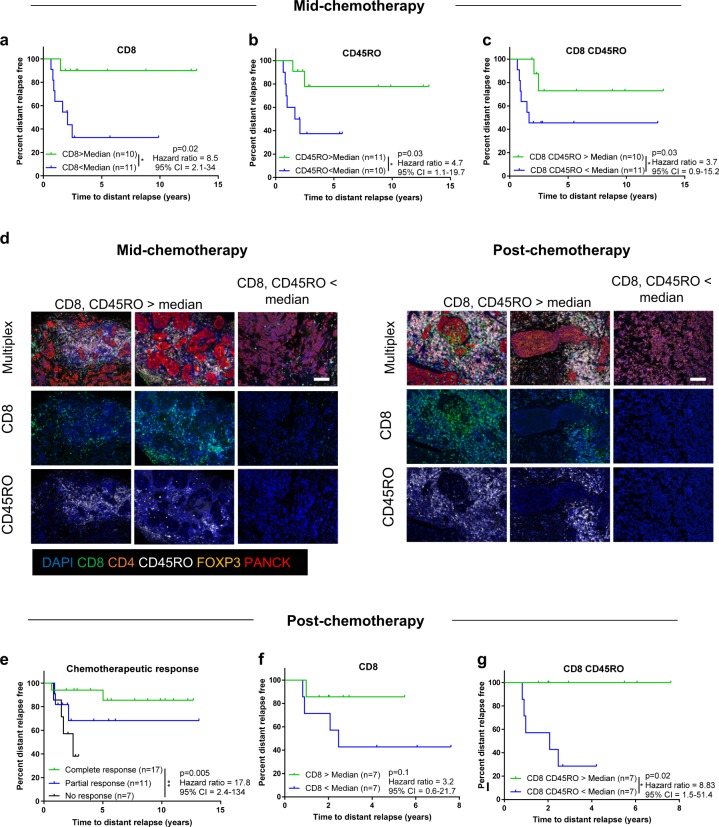
High proportions of antigen experienced CD8 T cells predict long-term survivors. Kaplan–Meier survival curve comparing distant relapse-free survival in TNBC patients mid-chemotherapy based on proportion of CD8^+^ T cells (**a**), CD45RO^+^ cells (**b**), or CD45RO^+^ CD8^+^ T cells (**c**) with groups divided by above or below the median (medians; **a** 10.93%; **b** 8.25%; **c** 3.4%). **d** Representative staining of TNBC primary tumors mid-chemotherapy and post-chemotherapy with CD8^+^ and CD45RO^+^ lymphocytes above or below the median. Sections measuring 3 μm were co-stained for expression of CD8 (green), CD4 (orange), CD45RO (white), FOXP3 (yellow), and PanCK (red). DAPI was used to visualize cell nuclei. Images are shown as multiplexed fluorescent images or single fluorescent images. Scale bars represent 200 μm. **e** Kaplan–Meier survival curve comparing distant relapse-free survival in TNBC patients who had a complete response, partial response, or no response to chemotherapy. Kaplan–Meier survival curve comparing distant relapse-free survival in TNBC patients post-chemotherapy based on proportion of CD8^+^ (**f**) and CD45RO^+^ CD8^+^ T cells (**g**) with groups divided by above or below the median (medians; **f** 6.5% ; **g** 1.63%). *p* values, hazard ratios, and confidence intervals calculated using a log-rank test (Mantel-Cox)

To test the link between immune infiltrate and metastasis-free survival, patients were stratified based on immune infiltrate and time to distant relapse. At mid-chemotherapy, the proportions of CD8^+^ T cells, CD45RO^+^ cells, and CD8^+^ CD45RO^+^ cells were closely linked to distant relapse (Fig. 2a–c). Patients who had a high proportion of these immune cell subsets mid-chemotherapy had reduced risk (hazard ratios of ~4) of metastatic relapse (Fig. 2a–c). Representative images highlight the differences in infiltrating immune cells between patients who had high or low proportions of CD8^+^ CD45RO^+^ cells at mid-chemotherapy assessment (Fig. 2d). Again, assessment of individual immune populations was superior to TIL score alone at predicting metastatic relapse both mid-chemotherapy and post-chemotherapy in this small cohort (Fig. 2; Supplementary Fig. 4k–n) supporting characterization of the specific nature of infiltrates in larger validation cohorts.

Post-cessation of treatment we confirmed a close association between complete chemotherapeutic response and prolonged relapse-free survival, with a partial or lack of response predicting patients with an increased risk of distant relapse (Fig. 2e). Although the proportion of CD8^+^ T cells (Fig. 2f) or CD45RO^+^ cells (Supplementary Fig. 4o) as single markers did not predict distant relapse-free survival post-chemotherapy, elevated tumor percentages of CD45RO^+^ CD8^+^ T cells predicted favorable patient outcome and extended metastasis-free survival (Fig. 2g, representative images in 2d), suggesting that post-chemotherapeutic induction of memory T cells is imperative in preventing distant relapse. Together this suggests that further characterization of TILs, in particular expression of CD45RO on CD8 T cells, offers important prognostic information during and after chemotherapy that may predict poor chemotherapeutic response long-term and highlight patients that may benefit from immune activating therapies. The observation that there was an overall decrease in the proportion of T cells infiltrating the tumors of patients who develop relapse (Supplementary Fig. 4i, j) suggested that these tumors were “non T-cell inflamed”.

### Tumor inherent IRF9 as a biomarker of metastasis-free survival

**Fig. 3:**
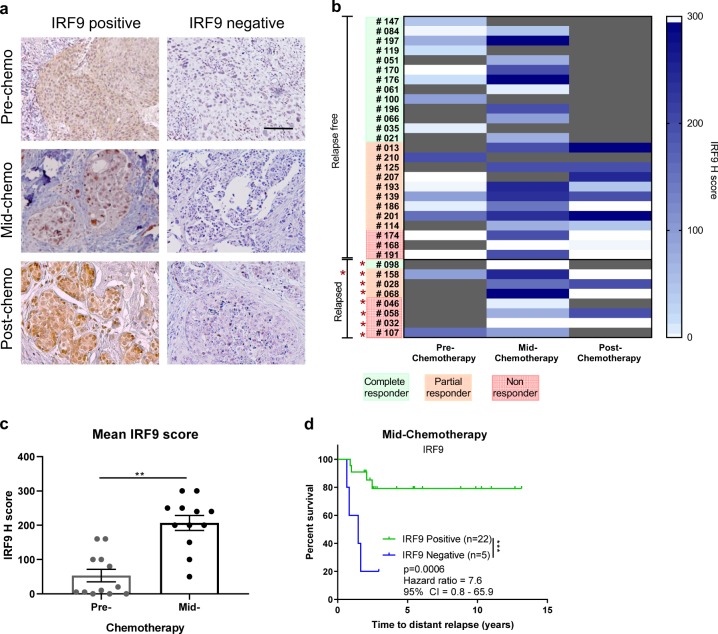
Loss of IRF9 predicts rapid metastatic relapse. **a** IRF9 expression in TNBC primary tumors pre-chemotherapy, mid-chemotherapy, and post-chemotherapy was evaluated by IHC. Tissues were stained using rabbit anti-IRF9 antibody (5 μg/ml), IRF9 expression visualized using DAB prior to nuclear counterstain with hematoxylin. Representative images were taken of primary tumors with high and low staining, scale bars represent 100 μm. **b** Heat map representing IRF9 H score in the tumor cells of TNBC primary tumors throughout chemotherapy. Gray represents no evaluable sample available. **c** IRF9 H score in sequential TNBC primary tumors pre-chemotherapy and mid-chemotherapy. Error bars represent SEM. *p < 0.05 using Mann–Whitney *U* test. **d** Kaplan–Meier curve comparing distant relapse-free survival in patients who had IRF9-positive tumors or IRF9-negative tumors (positive IRF9 is determined as *H* score > 20) mid-chemotherapy. *p* values, hazard ratios, and confidence intervals calculated using a log-rank test (Mantel-Cox)

As type I IFNs are implicated in the T-cell inflamed tumor and involved in immune cell activation,^24^ we aimed to characterize the prognostic and predictive potential of tumor inherent IFN status in the SETUP trial. To do this we interrogated the tumor expression of IRF9, a key transcription factor that is induced by IFN and forms part of the ISGF3 complex, responsible for stimulation of many IRGs—including IRF7. Immunohistochemical staining revealed heterogeneous expression of IRF9 across the SETUP trial cohort, with some tumors expressing high tumor IRF9 whilst others had low or undetectable levels (Fig. 3a). There was a clear increase in IRF9 mid-chemotherapy compared to baseline as represented by the increase in *H* score (Fig. 3b, c; Supplementary Fig. 5a). The expression of IRF9 mid-chemotherapy was associated with risk of distant relapse (Fig. 3d), whereby patients with low tumor IRF9 expression mid-chemotherapy were over seven times more likely to develop metastatic relapse than patients with high tumor IRF9 expression (Fig. 3d). The same trend was seen post-chemotherapy where lack of tumor IRF9 in partial or non-responders was associated with accelerated distant relapse (Supplementary Fig. 5b). All patients included in the trial had surgery post-chemotherapy and hence we also assessed IRF9 expression in the non-tumor epithelial tissue of complete responders. IRF9 epithelial expression was high in the complete responders (Supplementary Fig. 5c) and when time to distant relapse was analyzed including complete, partial, and non-responders, the loss of IRF9 did predict increased risk of rapid metastasis (Supplementary Fig. 5d). This finding suggests that IRF9 is a candidate prognostic biomarker, the loss of which predicts relapse in those patients who do not have a complete response to chemotherapy, those that are currently difficult to stratify.

### IRF9 expression correlates with active IFN signaling and a T_RM_ signature

**Fig. 4:**
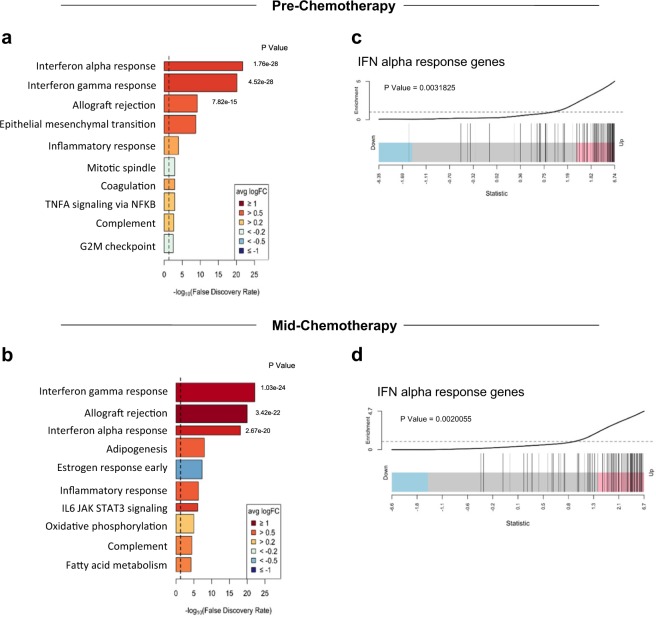
IRF9 reflects active IFN signaling pathways and a T_RM_ signature. Top ten most significant Hallmark gene sets enriched in IRF9-positive tumors compared to IRF9 negative tumors pre-chemotherapy (**a**) and mid-chemotherapy (**b**). Both pre-chemotherapy and mid-chemotherapy samples compared based on pre-chemotherapy IRF9 staining. Width of bars indicate relative number of genes in the gene set. IFNα response signature enrichment pre-chemotherapy (**c**) and mid-chemotherapy (**d**). Tumor samples from IRF9-positive patients (*n* *=* 5) compared with IRF9-negative patients (*n* *=* 8) (limma “roast” gene set test). Statistic on *x-*axis is the gene wise moderated *t*-statistic computed by limma, and vertical bars represent *t*-statistic for each gene in the gene set

To assess whether the tumor inherent expression of IRF9 reflected active IFN signaling pathways we performed whole-genome expression analysis on the same tissues derived from the SETUP trial. We analyzed the differences in gene expression profiles based on IRF9 baseline expression. Using competitive gene set testing, we found IRF9-positive tumors to be highly enriched for IFN alpha and gamma response gene sets pre-chemotherapy and mid-chemotherapy (Fig. 4a, b). The enrichment of type I IFN response genes in pre-chemotherapy and mid-chemotherapy samples based on basal IRF9 are illustrated on barcode plots (Fig. 4c, d). This suggests that IRF9 expression can be used as a surrogate marker of active IFN signaling in the TME at pre-chemotherapy or mid-chemotherapy time points, which may be driving an influx of specific immune populations. Given the numerous impacts of type I and II IFNs on immune activation it is plausible that IRF9 offers information beyond the general TIL infiltrate. This was evident by a lack of correlation between IRF9 *H* score and proportion of TILs or overall CD45RO^+ ^cells throughout chemotherapy (Supplementary Fig. 6a–f). However, IRF9 *H* score did correlate with the T_RM_ signature^17^ in our SETUP trial cohort (Supplementary Fig. 6g, h). As with the interferon response genes, the T_RM_ signature was enriched in our IRF9-positive tumors pre-chemotherapy and mid-chemotherapy (Supplementary Fig. 6g, h), suggesting the presence of T_RM_ cells in IRF9-positive tumors. Together, these data suggest that IRF9 is a surrogate marker for a T-cell inflamed tumor.

### Manipulation of the IFN pathway alters long-term sensitivity to chemotherapy

The human data suggested a close link between IFN signaling and chemotherapeutic response. To assess whether tumor inherent interferons can actually drive chemotherapeutic sensitivity and a hot TME, we enhanced IFN production and signaling in highly metastatic murine 4T1.2 TNBC cells via enforced expression of the master regulator of IFN production—IRF7 (a target of IRF9). Enforced expression of IRF7 in 4T1.2 cells has previously been shown to restore hundreds of IRGs^29^ as elevated expression leads to direct self-phosphorylation and IFN production. As IRF9 requires complexing with STAT1 and STAT2 for formation of the ISGF3 complex, its elevation alone does not ensure elevated production of type I IFN. pMSCV driven Irf7 overexpression in 4T1.2 cells (4T1.2 IRF7 OE) was confirmed at the transcriptional level (Supplementary Fig. 7a) and enhanced pathway signaling was validated via assessment of IFNα production (Supplementary Fig. 7b) when compared to control cells transduced with base vector (4T1.2 BV). In order to ensure that Irf7 levels did not surpass physiologically relevant levels, Irf7 transcript was compared between 4T1.2 IRF7 OE and cell lines of varying metastatic potential (Supplementary Fig. 7c). The 4T1.2 IRF7 OE line expression of Irf7 did not exceed that of weakly metastatic EO771 and 67NR cell lines and was comparable to the 4T1 cell line (Supplementary Fig. 7c) suggesting Irf7 elevation was not at nonphysiological levels.

**Fig. 5:**
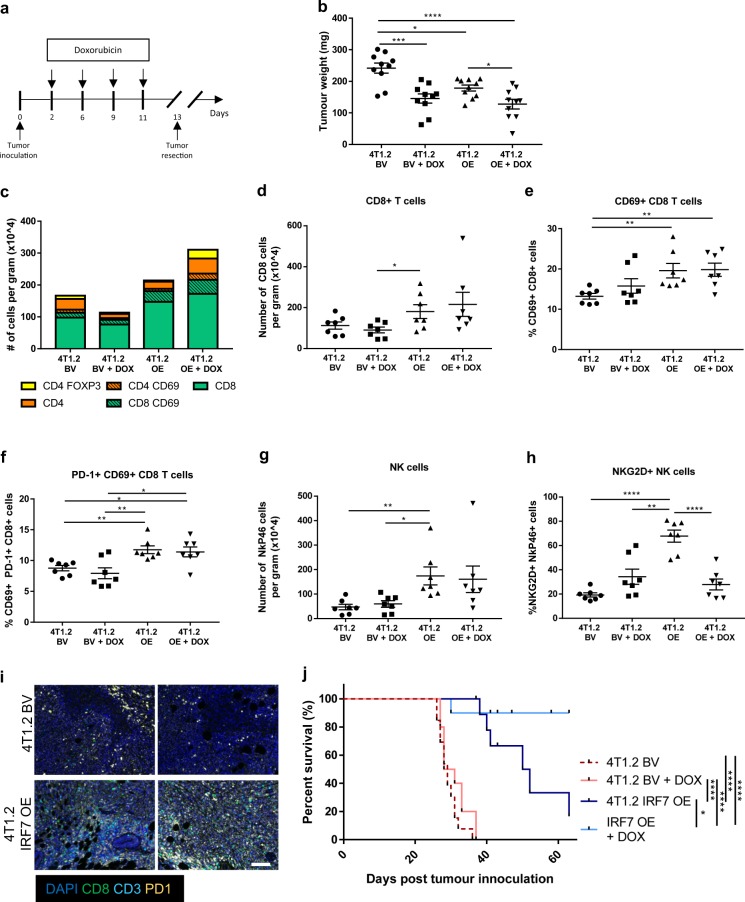
Enforced interferon signaling promotes enhanced immune activation and chemotherapeutic sensitivity. **a** Treatment protocol for doxorubicin therapy (4 mg/kg) of BALB/C mice injected with 1 × 10^5^ 4T1.2 BV or 4T1.2 IRF7 OE cells IMFP. **b** Tumor weight (mg) 13 days post tumor cell inoculation (*n* *=* 10 per group). **c** Bar graph representing 4T1.2 BV or 4T1.2 IRF7 OE primary tumor infiltrates in treated and untreated mice, showing number of CD8^+^, CD8^+^ CD69^+^, CD4^+^ CD69^+^, CD4^+^, and CD4^+^ FOXP3^+^ cells per gram determined via flow cytometry (*n* = 7 per group). Total number of CD8^+^ T cells per gram (**d**) and proportion of CD8^+^ T cells expressing CD69 (**e**), and CD69/PD-1 (**f**) isolated from the primary tumor (*n* *=* 7 per group). Total number of NKp46^+^ TCRβ^−^ lymphocytes per gram (**g**) and proportion of NKp46^+^ lymphocytes expressing NKG2D (**h**) isolated from the primary tumor (*n* *=* 7 per group). **i** Representative images of 4T1.2 BV or IRF7 OE primary tumors from saline treated mice. Sections measuring 3 μm were co-stained for expression of CD8 (green), CD3 (cyan), and PD-1 (yellow) followed by counterstain using DAPI to visualize cell nuclei. Images are shown as multiplexed fluorescent images. Scale bars represent 200 μm. **j** Kaplan–Meier curve comparing metastasis-free survival in mice bearing 4T1.2 BV or 4T1.2 IRF7 OE cells treated with saline or doxorubicin (*n* *=* 10 per group). Mice excluded due to primary tumor regrowth are indicated with a black dash. Error bars represent SEM. *p < 0.05; **p < 0.01*;* ***p *<* 0.001; ****p *<* 0.0001 using students *t* test or log-rank test (survival analysis)

Tumor cells (4T1.2 BV and IRF7 OE) were injected into the fourth mammary gland of Balb/c mice. To mimic the neoadjuvant clinical treatment setting, we administered doxorubicin or saline post tumor inoculation and ceased treatment prior to primary tumor removal (Fig. 5a). Analysis of primary tumor weight at resection revealed a moderate 4T1.2-BV tumor response to doxorubicin alone (Fig. 5b). Tumors derived from mice bearing 4T1.2 IRF7 OE cells were smaller than those bearing 4T1.2 BV, and tumor size was further reduced upon doxorubicin treatment (Fig. 5b). Importantly, this difference was not due to an inherent difference in proliferative rate in vitro (Supplementary Fig. 7d). TIL interrogation in dissociated tumors revealed a difference in the immune landscape between 4T1.2 BV tumors and 4T1.2 IRF7 OE tumors, with CD8^+^ T cells being the most predominant infiltrating lymphocyte identified (Fig. 5c; Supplementary Fig. 7e). Quantitation of immune cell numbers revealed elevated CD8^+^ T cells in 4T1.2 IRF7 OE tumors (Fig. 5d). The CD8^+^ T cells infiltrating 4T1.2 IRF7 OE tumors (±doxorubicin) were also more activated, with increased CD69^+^ CD8^+^ T cells (Fig. 5e) and PD-1^+^ CD69^+^ CD8^+^ T cells (Fig. 5f) compared to 4T1.2 BV tumors. A similar phenotype was also observed in innate immune cells, with elevated numbers of NK cells infiltrating 4T1.2 IRF7 OE tumors compared to 4T1.2 BV tumors (Fig. 5g; Supplementary Fig. 7e) and increased NKG2D^+^ NK cells in the 4T1.2 IRF7 OE tumors compared to all other groups (Fig. 5h). Multiplex IHC confirmed the increase in the general CD3^+^ and CD8^+^ T cell infiltrate along with PD-1, a known interferon stimulated gene, in the 4T1.2 IRF7 OE tumors compared to the 4T1.2 BV (Fig. 5i). Thus, enforced tumor expression of the master regulator of type I IFN signaling, IRF7, is sufficient to increase the accumulation and activation of tumor infiltrating lymphocytes, in particular, activated CD8^+^ T cells and NK cells. To test whether the enhanced immune activation led to prolonged metastasis-free survival post-chemotherapy and primary tumor resection, mice were individually assessed for signs of metastatic distress and groups were compared (Fig. 5j). Although we observed an impact on primary tumor growth, doxorubicin alone had no impact on metastasis-free survival in 4T1.2 BV tumor bearing mice (Fig. 5j, median survival 29 and 29.5 days). In contrast, enforced expression of IRF7 led to prolonged survival (Fig. 5j, median survival 51 days) and an enhanced response to doxorubicin therapy, such that only one mouse succumbed to metastasis by experimental endpoint (Fig. 5j, median survival undefined). Together, these data support tumor inherent type I IFNs as immune activators and important factors in chemotherapeutic response.

The observation that tumor inherent type I IFN signaling may be driving immune chemotherapeutic response and anti-tumor immunity led us to consider the potential reversibility of IRF9/IFN loss in human breast tumors, as a possible means for enhanced therapeutic response and prolonged survival. To assess this, we used a panel of human breast cancer cell lines and as with our observed expression in primary tumor tissues, the expression of IRF9 and IRF7 was heterogeneous across cell lines and subtypes (Supplementary Fig. 8a, b). Treatment of these cell lines with IFNα or the toll-like receptor (TLR) agonist, poly (I:C) was sufficient to enhance IRF9 and IRF7 expression in both ER^+^ (Supplementary Fig. 8c, f) and TNBC cell lines (Supplementary Fig. 8d, e, g, h). Chemotherapy had little impact on IRF9 and IRF7 expression in an in vitro setting. These data suggest that dampened tumor inherent IFN signaling is reversible and can be restored via stimulation of the pathway with IFN or indirectly via a type I IFN inducer.

### Loss of IRF9 expression predicts poor prognosis in TNBC

**Fig. 6:**
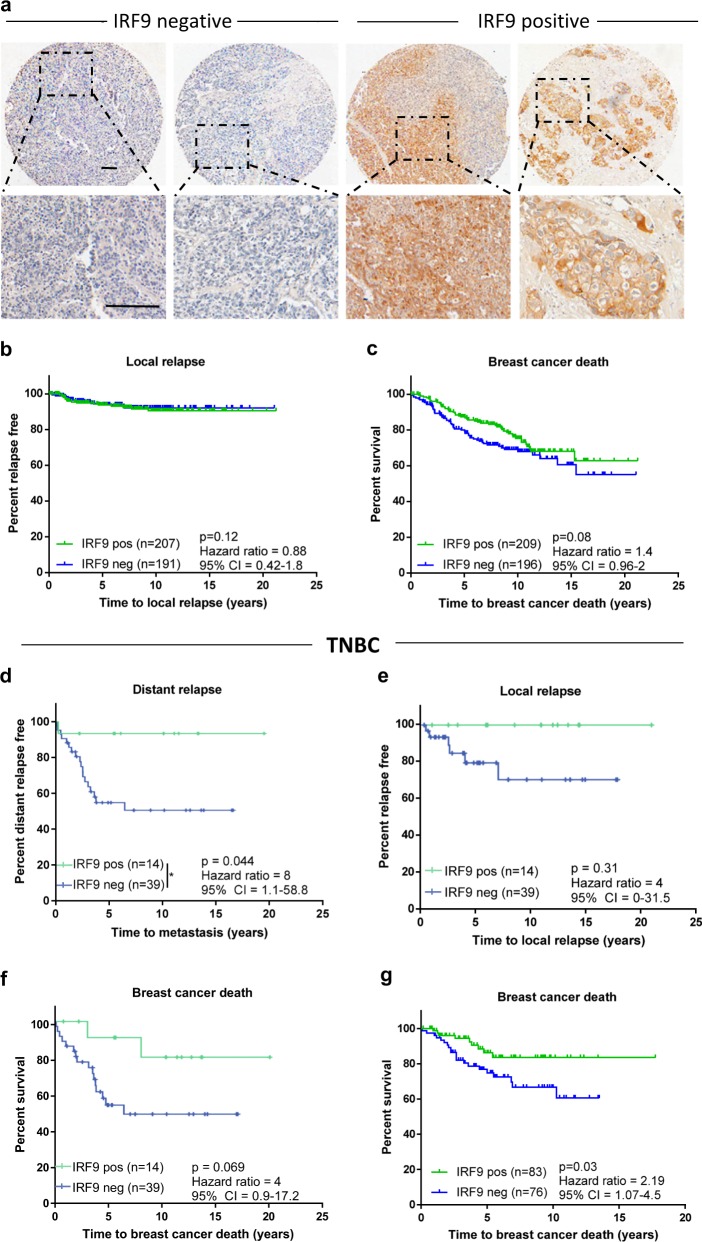
Loss of IRF9 expression predicts poor prognosis in TNBC. **a** IRF9 expression in primary breast cancer tumors pre-chemotherapy was evaluated by IHC. Tissues were stained using rabbit anti-IRF9 antibody (5 μg/ml), IRF9 expression visualized using DAB prior to nuclear counterstain with hematoxylin. Representative images were taken of primary tumors with high and low staining, scale bars represent 100 μm. Kaplan–Meier survival curves comparing time to local relapse (**b**) and breast cancer-specific death (**c**) in all breast cancer subtypes based on positive- or negative-IRF9 expression. Kaplan–Meier survival curves comparing time to metastasis (**d**), time to local relapse (**e**), and time to breast cancer death (**f**) in TNBC patients who had positive- and negative-IRF9 expression. **g** Kaplan–Meier survival curve comparing time to breast cancer related death in an independent TNBC cohort in patients who had positive or negative IRF9 expression. *p* values, hazard ratios, and confidence intervals calculated using a log-rank test (Mantel-Cox)

In order to independently validate IRF9 as a prognostic marker, we analyzed baseline IRF9 expression in an adjuvant cohort of 414 breast cancer patients of mixed breast cancer subtypes (ER^±^  luminal A and B, HER2^+^, and TNBC) where chemotherapy was administered subsequent to tumor resection (Supplementary Table 2). As with the SETUP trial, staining was homogeneous in individual tumors and heterogeneous across patients, with some tumors expressing high tumor IRF9, while others had low or undetectable levels (Fig. 6a). When IRF9 expression was interrogated across all breast cancer subtypes there was no association with time to local relapse (Fig. 6b) or breast cancer death (Fig. 6c). However, analysis of the TNBC subtype revealed that IRF9 was a prognostic biomarker in pre-chemotherapy samples, the loss of which predicted an eightfold increased risk of distant metastasis (Fig. 6d). There was also a clear trend between high IRF9 expression and a decreased risk of local relapse and breast cancer-specific death, yet this was not significant, likely due to the size of this cohort (Fig. 6e, f). Multivariate analysis confirmed IRF9 as an independent prognostic marker of risk of distant metastasis in this cohort (Supplementary Table 2). We expanded this to a larger independent TNBC-specific cohort of 159 patients (Supplementary Table 3) and confirmed expression of IRF9 as a predictor of prolonged breast cancer-specific survival (Fig. 6g). Based on IHC subtyping (CK5/6, EGFR expression) we further segmented TNBC into basal like breast cancer (BLBC, CK5/6^+^, and/or EGFR^+^) and non-BLBC (CK5/6^−^ EGFR^−^). There was no difference in IRF9 *H* score between BLBC and non-BLBC (Supplementary Fig. 8h) and analysis of IRF9 expression specifically in the BLBC subtype confirmed that high IRF9 expression was associated with a 2.5 times reduced risk of breast cancer death compared to patients with IRF9-negative tumors (Supplementary Fig. 8i). Together, our data support IRF9 as a prognostic marker in general TNBC and also BLBC.

## Discussion

This study highlights tumor inherent IFNs as key factors in patient outcome post-chemotherapy. Here, we demonstrate that tumor-inherent IRF9 is an important biomarker that reflects both active IFN signaling and a subsequent T_RM_ signature, indicative of a tumor-targeted T-cell memory response. Furthermore, we showed that it is tumor inherent IFN signaling which drives an immune reactive TME and metastasis-specific chemotherapeutic sensitivity using murine models of TNBC.

Our investigation using a cohort of TNBC patients who received neoadjuvant chemotherapy and underwent sequential biopsies (SETUP trial) emphasizes the important prognostic information that can be gathered throughout chemotherapy. Although neoadjuvant chemotherapy offers important prognostic information based on the final pathological response of a tumor, with a complete response being closely linked to a favorable prognosis [*8 and data herein*] markers are needed to discriminate non- or partial-responders that are most at risk of metastatic relapse. Our data have revealed markers that allow such prognostic assessment pre-, mid-, and prior to cessation of chemotherapy, potentially allowing therapeutic opportunities earlier which could impact patient mortality. Importantly, IRF9 expression allowed stratification of partial or non-responders based on risk of metastatic relapse. Not only does this work support neoadjuvant therapy for the discovery and development of biomarkers, studies in multiple murine TNBC models have also shown improved outcomes to alternative therapies when therapy is given in the neoadjuvant setting as opposed to adjuvant.^33,34^ Despite previous reports showing no safety issues with multiple biopsies, it is not currently standard of care to assess the tumor at multiple times throughout therapy.^35^ However, our results mid-chemotherapy suggest that implementation of sequential biopsies into the clinic could offer important information about tumor “heat” that will likely contribute to individualized treatment approaches for TNBC patients.

Current efforts to profile tumor heat in the context of the immune landscape have ranged from basic TIL score to single-cell gene expression profiling of immune subsets present in the TME. High TILs in TNBC is associated with better outcome post-chemotherapy^36,37^ however the numerical cut-offs used in multiple studies varied, suggesting a lack of consensus when separating TIL high and TIL low patients^38^ and likely contributing to the lack of incorporation of a TIL score in routine pathological assessment of TNBC. Further characterization of immune cells has revealed the unique role that different immune subsets play in patient prognosis with CD8^+^ T cells remaining at the forefront of interest, where their enhanced infiltration has been associated with a good outcome in many types of cancer.^12,15,18,39,40^ In TNBC, efforts to profile CD8^+^ T cells revealed that a T_RM_ phenotype, by gene expression profiling, was associated with improved survival outcomes.^17^ The same gene expression profile when applied to melanoma datasets could distinguish between responders and non-responders to immunotherapy pre-treatment^17^ indicating that the quality of the T-cell infiltrate is important. Our results support this notion whereby it was the presence of antigen experienced cells (CD8^+^ CD45RO^+^) that associated with distant relapse-free survival. These results highlight that the implementation of multispectral IHC into the clinic may offer prognostic information, though there is a clear need for a validation cohort given that patients were stratified purely based on arbitrary cut-offs determined using a small cohort. Use of the SETUP trial did not uncover altered proportions of immune cell subtypes during chemotherapy, these results suggest that alternatives to chemotherapy should be considered to bolster immune infiltration in patients with cold tumors.

The T-cell inflamed tumor, identified via gene expression profiling, has been associated with improved responses to therapy in melanoma.^18^ Our investigation into what drives responses in TNBC has revealed similar findings, where it is not only the immune cells present that drive responses but also the presence of key cytokines, the type I and II IFNs. Using basic IHC, we show that lack of the type I IFN transcription factor IRF9 in tumor cells can predict metastatic relapse. Whilst we have previously shown that loss of an entire IFN gene set occurs in bone metastasis^29^ and that host IFNAR is required for effective anti-tumor immunity,^26^ this is the first report of a single IFN biomarker in the primary tumor that can predict risk of metastasis and reflects active IFN signaling and a T_RM_ signature. We were also able to demonstrate the ability of IRF9 to predict long-term survival in an adjuvant TNBC cohort, suggesting it is a biomarker pre-therapy and mid-therapy. Importantly, unlike immune infiltrates where clear thresholds are difficult to assign for diagnostics across independent cohorts we were able to define clear cut offs for IRF9 based on *H* score that we believe could be applied to individual patients going forward. Previous findings by our laboratory^29^ have highlighted that in other subtypes, including ER^+^ breast cancer, loss of type I IFN regulators can occur post tumor dissemination to bone, reducing the impact of IRF7 and IRF9 as primary tumor prognostic markers in ER +ve disease. Further studies into the effect of estrogen signaling and targeted therapies on tumor intrinsic interferon signaling is required to determine the mechanisms of subtype specific regulation of this pathway in breast cancer.

An important finding was the induction of IRF9 expression mid-chemotherapy in some patients, suggesting that increased expression during and following chemotherapy cessation is linked to long-term response. Increased IRF9 mid-chemotherapy and post-chemotherapy could be due to release of cytoplasmic DNA-inducing IFN signaling, which may trigger events leading to immunogenic cell death (ICD). Previous studies have shown that both anthracycline-based chemotherapy and radiotherapy can induce ICD, resulting in the upregulation of IRGs.^30,41,42^ The induction of IRGs postradiotherapy and chemotherapy has also been linked to improved response rates, suggesting the induction of an immune response post-chemotherapy via the upregulation of type I IFN signaling postnucleic acid sensing is required for long-term benefit.^30,42^ This data, together with our results, suggest IRF9 provides a good readout of therapeutic response and overall survival after chemotherapy treatment.

While we have previously shown both systemic and tumor induction of type I IFN can improve metastasis-free survival in murine TNBC models,^29,30,33^ here, we have shown that it is type I IFN signaling that really drives lymphocyte infiltration and chemotherapeutic response. Although we were not able to get access to TNBC patients that did not receive chemotherapy and therefore cannot definitively state that IRF9 predicts long-term chemotherapeutic outcome, our genetically altered mouse model does suggest that tumor inherent IFN status does predict improved outcomes in chemotherapy naïve and treated mice. A link between type I IFN and immunotherapy responses has also been identified in TNBC and melanoma, where use of an IFN inducer was able to sensitize mice to checkpoint-based immunotherapy.^33,43^ Our observation that type I IFN or IFN inducers stimulate IRF9 expression in human TNBC cell lines further supports the implementation of IFN-based therapeutics into the clinic. The use of viral mimetics, such as TLR or STING agonizts, are actively being explored in a multitude of clinical trials based on preclinical data in a number of malignancies.^44,45^ Our data suggests that IRF9 may serve as an important biomarker to identify patients who could benefit from such approaches either pre-, mid-, or post-chemotherapy.

Our findings indicate IRF9 as a robust biomarker that can reflect overall tumor heat and metastasis-free survival in TNBC. This may allow stratification of patients into alternative therapy groups early, as patients with tumors expressing IRF9 are likely to do well on standard of care chemotherapy, while those lacking IRF9 may require further intervention that could be implemented prior to cessation of cytotoxic agents. This supports expanding current trials incorporating IFN inducers into the neoadjuvant TNBC treatment setting, with IRF9 as a candidate biomarker for patient stratification.

## Materials and methods

### Patient cohorts

Three independent cohorts were used for this study. The SETUP trial tumor samples were obtained from Monash and Peninsula Health. Patients with locally advanced breast cancer, deemed suitable for neoadjuvant cytotoxic chemotherapy, consented for the collection of imaging data and biological specimens for biomarker analysis (SETUP study) and were enrolled into a phase III randomized controlled neoadjuvant clinical trial which was approved by the Human Ethics Research Committee and the Monash Medical Centre (ANZCTR.org.au clinical trials identifier: ACTRN12605000588695; HREC/SETUP/03169A), all patients provided written consent to participate. A total of 35 TNBC cases were acquired for analysis. Due to either insufficient tissue remaining after standard histological assessment, sampling issues or lack of remaining tumor total number of patients analyzed at each time point differed (Supplementary Fig. 1), as such time points were analyzed independently. All patients enrolled in the trial received 3 months of fluorouracil, epirubicin, and cyclophosphamide, and three months of docetaxel. Tumor size was monitored via PET imaging and CT scanning. The primary endpoint of this study was pathological response at time of surgical resection with secondary outcome being time to distant relapse. Baseline data collected, included patient demographics and prognostic variables, as well as clinical outcomes such as response rates, progression-free survival and overall survival. Patients were categorized as complete responders where there was no evidence of remaining tumor post-chemotherapy (confirmed by PET, CT, and histology), partial responders where a reduction in tumor size from baseline was observed, and non-responders where tumor size was unchanged or progressed during chemotherapy. The St. Vincent’s Hospital mixed subtype cohort has previously been described,^46^ as has The Royal Prince Alfred Hospital (RPAH) cohort^47^ (HREC/15/RPAH/531, X15-0388) (HREC/08/CIPHS/62).

### Histology and IHC

Prior to paraffin embedding and sectioning, all tissues were fixed in 10% neutral buffered formalin (NBF). For morphological tissue analysis and TIL scoring Hematoxylin and Eosin (H&E) staining was performed. For evaluation of IRF9 expression, tissues were subjected to heat induced epitope retrieval in a pressure cooker (110 °C for 5 min) in citrate buffer (pH 6) before incubating with anti-IRF9 (ab51639; 5 μg/ml, Abcam) at 4 °C overnight. Tissues were then incubated with enzyme conjugated secondary antibody followed by incubation with Avidin/Biotinylated enzyme complex (ABC; Vectastain) and visualization with diaminobenzidine (DAB; Vectastain) color development system. Tissues were counterstained using hematoxylin. Scoring of IRF9 was performed by a trained pathologist, tissues were given a *H* score based on percentage positivity and intensity as per standard scoring guidelines.^48^

### Multiplex IHC

Human TILs were characterized using CD4, CD8, CD45RO, FOXP3, and PanCK in TNBC patient biopsies and tissue micro arrays using the Opal 7-color tumor infiltrating lymphocyte kit according to the manufacturer’s protocol (OP7TL3001KT; PerkinElmer). Primary antibody concentrations were optimized (CD4, 1:150; CD8, 1:200; CD45RO, 1:150; FOXP3, 1:100, PanCK, 1:500) and protocol modified to include 1 h primary antibody incubations. After all rounds of staining, tissues were counterstained with DAPI (PerkinElmer) and mounted in Vectashield hard set mounting medium (Vector). Whole slide scans and multispectral imaging of sections was undertaken using the VECTRA 3.0 (PerkinElmer) at ×20 magnification. Image analysis and phenotyping was undertaken using the inForm software (PerkinElmer) following tissue segmentation into tumor and stromal areas (Supplementary Fig. 1). Graphs were generated using GraphPad prism (version 7). Survival curves were compared using log-rank (Mantel-Cox), other graphs were compared using Mann–Whitney *t* tests (two-tailed).

Murine immune cells were visualized using CD3, CD8, and PD-1 via the OPAL method using the seven-color manual IHC kit (PerkinElmer; NEL811001KT). Murine primary tumors were fixed in 10% NBF prior to paraffin embedding and sectioning. Staining was performed as per manufacturer’s protocol with the exception of 30 min blocking and secondary antibody incubations. Primary antibodies for CD8 (4SM15, 5 μg/ml; ThermoFisher), CD3 (SP7, 1/125; Abcam), and PD-1 (2 μg/ml; Proteintech) were incubated for 1 h at RT. Donkey anti-rabbit HRP (Chemicon AP182P; 1:2000) or donkey anti-rat HRP (Chemicon AP189P; 1:2000) secondary antibodies were utilized. After all rounds of staining, including DAPI, tissues were mounted using Vectashield hard set mounting medium (Vector). Whole-slide scans and multispectral imaging of sections was undertaken as described above.

### TIL quantification and profiling

Quantification of stromal TILs (percentage) in the SETUP trial was performed manually and blindly by a trained pathologist using whole slide scanned H&E aperio images according to the published protocol.^36^ Percentage of TILs is the fractional area of stroma infiltrated by TILs. Quantification of stromal TILs was also performed post multiplex IHC using inForm software (PerkinElmer) where stromal cells were counted after the tissue was segmented into stromal and tumor areas.

### RNA-seq

RNA was prepared from patient tissue using the AllPrep DNA/RNA/miRNA Universal kit (Qiagen #80224) as per manufacturer’s protocol. The TissueLyser II was used for tissue homogenization. On column DNA digestion was performed to ensure pure RNA was isolated. Samples were quantified and quality checked using Nanodrop, Qubit® 2.0 Fluorometer. RNA integrity was assessed using Agilent 2100 Bioanalyzer.

RNA-seq was performed in 2 batches, with 35 samples and 47 samples, respectively. Sequencing was performed on the Illumina Hiseq2500 (125 bp paired-end reads) on a high-output mode. In the first batch, total RNA-seq libraries were prepared with 250 ng of total RNA using the Illumina® TruSeq® Stranded total RNA Sample Preparation kits as per manufacturer’s protocol. RNA fragmentation time was adjusted to avoid over fragmentation for the low-quality samples (RIN value lower than 6). Final library samples were multiplexed and spread across eight lanes, with an average of 50 million paired-end reads per sample. In the second batch, mRNAseq libraries were prepared with 500 ng of total RNA using the Illumina® TruSeq® Stranded mRNA Sample Preparation Kits as per manufacturer’s protocol. The final library samples were multiplexed and spread across four lanes, with an average of 20 million paired-end reads per sample.

### Transcriptomic analysis

Raw reads from RNA-Seq were firstly assessed by FastQC and FastQ screen, then filtered using FastQ-mct (Github repository). Filtered reads were aligned to the human reference genome GRCh38 using STAR software version 2.4.2.^49^ Feature count was obtained using RSEM version 1.2.21.^50^ Counts were processed using the limma package.^51^ Genes were analyzed if they were detected in at least three samples, with at least one count per million. Normalization factors were determined with the edgeR calcNormFactors function using the TMM method.^52^ Gene expression between patient samples with positive and negative IRF expression pre-chemotherapy (positive expression was defined as a *H* score > 20) was compared pre-chemotherapy, mid-chemotherapy, and post-chemotherapy. Counts were converted to weighted log counts per million expression values using the voom with quality weights^53^ function. A batch effect, corresponding to sample library preparation, was incorporated into the design matrix. Patient effect was incorporated as a blocking factor in the linear model, with intersubject correlation obtained using the duplicate Correlation function,^54^ and moderated *t*-statistics were calculated using the eBayes function.^55^ Gene set testing was performed using the camera PR function^56^ with the Hallmark gene set collection^57^ from the molecular signatures database.^58^ Barcode plots were created for the Hallmark interferon alpha response gene set and a tissue resident memory T cell signature.^17^ *p* Values for gene set upregulation were obtained using the roast function,^59^ with 999,999 rotations.

### TNBC models

The highly metastatic 4T1.2 subclone of the 4T1 line was derived in and sourced from Prof. Robin Anderson’s laboratory.^60,61^ The pMSCV-IRES-mCherry retroviral expression vector (Addgene) was used to enforce Irf7 expression. Empty plasmid or plasmid containing Irf7 construct was transfected into phoenix-eco packaging cells using lipofectamine (Invitrogen). Conditioned media was filtered and incubated with target 4T1.2 cells. Cells were then fluorescence activated cell sorted (FACS) for mCherry expression as single cells and multiple clones with confirmed expression were then pooled for use. Both the 4T1.2 base vector (4T1.2 BV) and 4T1.2 IRF7 overexpression (4T1.2 IRF7 OE) cell lines were cultured in α-MEM (5% FBS). All cell lines (human/murine) were passaged using EDTA (0.01% w/v in PBS) and cultured for no longer than 4 weeks. Tumor lines were verified to be *mycoplasma* negative by the Victorian Infectious Diseases References Laboratory (Melbourne, Vic, Australia).

Balb/c mice were obtained from the Walter and Eliza Hall Institute of Medical Research (Melbourne, Vic, Australia). Mice were used between the ages of 8–12 weeks. All experiments were approved by the La Trobe Animal Ethics Committee.

### In vivo treatment and survival analysis

For in vivo experiments, 1 × 10^5^ cells (4T1.2 BV or 4T1.2 IRF7 OE) were injected in PBS (20 µL) into the fourth mammary fat pad (IMFP) on day 0. Doxorubicin hydrochloride (doxorubicin, dox) at 4 mg/kg or saline was administered intravenously (IV) twice weekly from days 2–11 post tumor cell inoculation. Mammary tumors were resected at day 13 post tumor cell inoculation and weighed. Mice were sacrificed individually upon signs of metastatic distress and lung metastasis confirmed via histology. Survival curves were generated using GraphPad Prism (version 7) and compared via log-rank (Mantel-Cox) test.

### Flow cytometry analysis

For analysis of tumor infiltrating lymphocytes, a single-cell suspension of primary tumors was obtained using mechanical and enzymatic digestion (1 mg/ml collagenase I (Sigma) and 30 μg/ml DNAse I (Sigma) at 37 °C) before red blood cell lysis (155 mM NH_4_Cl, 10 mM KHCO_3_, 0.1 mM EDTA, pH 7.3). Single-cell suspension was then stained with panels of antibodies (dilution 1/300); CD8a-PE-Cy7 (53–6.7), CD4-APC-Cy7 (GK1.5), CD69-APC (H1.2F3), NKP46-A700 (29A1.4), TCRβ-FITC (H57-597), CD279-PE (J43) (all from BD Biosciences) and NKG2D-PE-Cy7 (CX5) (eBioscience) before being subject to flow cytometry analysis using the FACS ARIA III (BD Biosciences) and data analyzed using Flowjo software (Tree star). Data have either been normalized to tumor weights or represented as percentage of lymphocytes as indicated.

### Reporting summary

Further information on research design is available in the Nature Research Reporting Summary linked to this article.

## Supplementary information

Reporting Summary

Supplementary methods and figures

## Data Availability

The authors declare that the data supporting the findings of this study are available within the paper (and its supplementary information files).

## Supplementary information

**Supplementary Information** accompanies the paper on the *npj Precision Oncology* website (10.1038/s41698-019-0093-2).
