# Supplementary material for: Tumor inherent interferon regulators as biomarkers of long-term chemotherapeutic response in TNBC
Source: NPJ Precis Oncol. 2019 Aug 29;3:21. doi: 10.1038/s41698-019-0093-2 (PMC6715634; doi:10.1038/s41698-019-0093-2)
Supplement: Supplementary file 2 — Supplementary methods and figures [file 41698_2019_93_MOESM2_ESM.pdf]

Supplementary methods

Cell lines

Human breast cancer cell lines were obtained from ATCC and DMSZ. Cell lines were grown in DMEM supplemented with 10% FBS (Gibco) (CAL-120, MDA-MB-231, MDA-MB-468) or RPMI supplemented with 10% FBS + 5µg/ml insulin (Sigma) (MDA-MB-453, HCC70, MCF7).

IFNα ELISA

IFNα ELISA was performed using standard molecular biology techniques. Capture antibody, clone RMMA-1 was used at 1/500 (0.16µg/ml; PBL Interferon source) prior to detection antibody (1/500 rabbit polyclonal mouse IFNα 32100-1; 4µg/ml; PBL Interferon source). Cells were seeded and transfected with vehicle or poly (I:C) (10µg/ml) for 24 hours prior.

PCR

Cell lines were treated with IFNα (1000 IU/mL), doxorubicin hydrochloride (20nM) or transfected with poly (I:C) (10µg/ml) for 24-48 hours prior to pelleting. RNA was extracted from cells using the Trizol method (1) and converted into cDNA using the iScript cDNA synthesis kit (BioRad). Real time (RT) qPCR was used to quantify murine and human IRF9 and IRF7 transcript expression via CFX384 (BioRad). Gene expression (arbitrary units; AU) was based on the quantification cycle (C_q) for gene of interest relative to house keeper genes (murine, Hprt; human, RPS18) and displayed as relative transcript abundance.

Sulforhodamine B (SRB) proliferation assay

In vitro proliferation was assessed using a sulforhodamine B –binding assay as previously described (2) with the 4T1.2 BV and IRF7 OE cells over six days with a starting cell number of 500 cells.

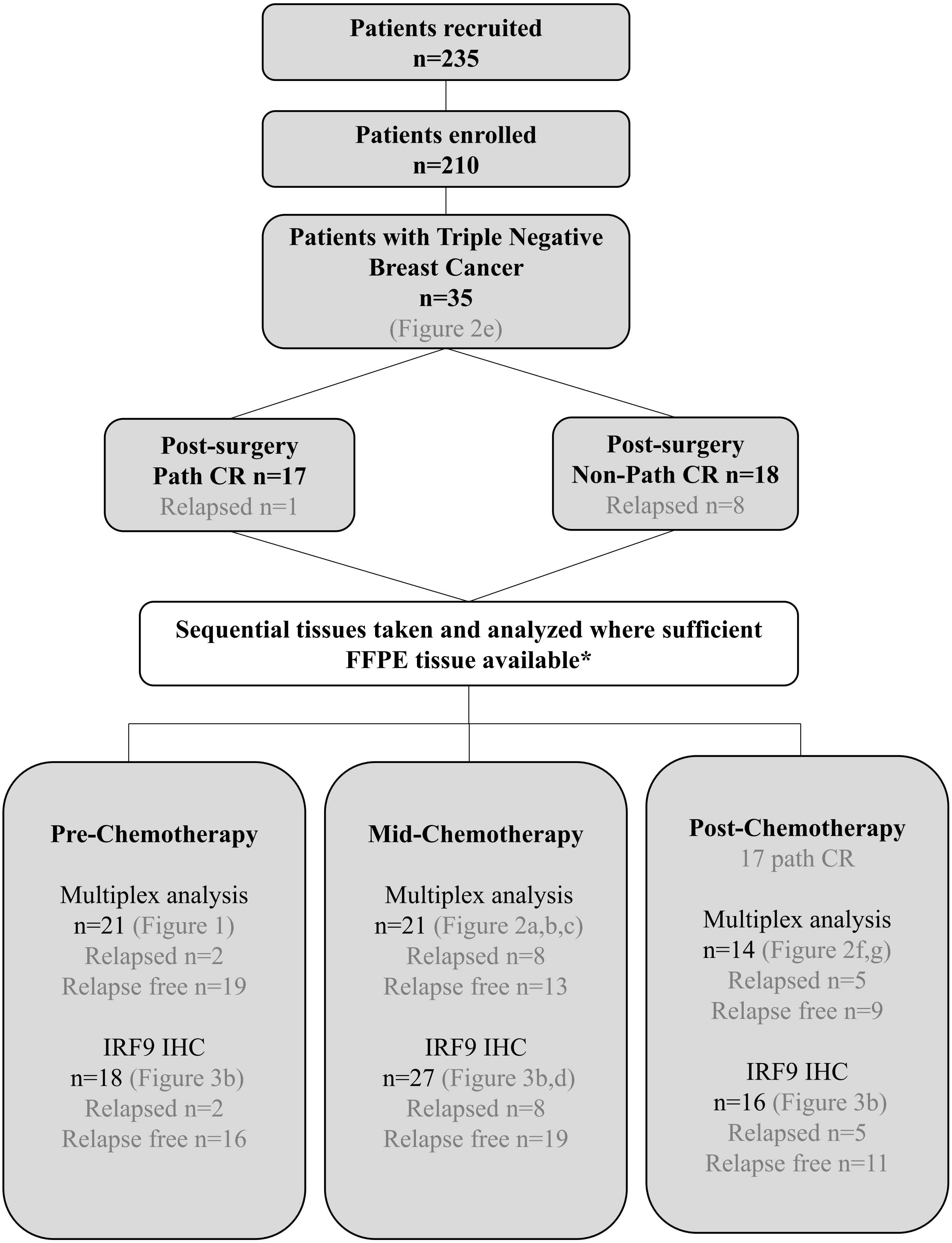

*Each time point was analyzed independently, 1 sample per patient

Supplementary Figure 1

Supplementary figure 1: REMARK diagram

REMARK diagram for the SETUP trial indicating the number of patients enrolled and number of specimens available for evaluation at each time point. Corresponding figures at each time point are indicated in the diagram. Samples were excluded due to tissue degradation or loss during the multiple rounds of staining.

Supplementary Table 1: SETUP trial patient characteristics

	SETUP Trial Cohort	
	Number	*Percent*
**Total**	35	
**Age**		
<45	16	45.71%
45-55	9	25.71%
55-65	7	20%
>65	3	8.57%
**Grade**		
1	0	0.00%
2	4	11.43%
3	31	88.57%
**Tumour size**		
T1	0	0.00%
T2	13	37.14%
T3	22	62.86%
**Response to chemotherapy**		
Complete	17	48.57%
Moderate	11	31.43%
None	7	20.00%
**IRF9 status**		
**Pre-Chemotherapy**		
Negative	9	28.57%
Positive	9	25.71%
Unknown	17	45.7%
**Mid-Chemotherapy**		
Negative	5	14.29%
Positive	22	62.86%
Unknown	8	22.86%
**Post-Chemotherapy**		
Negative	8	22.86%
Positive	23	65.71%
Unknown	4	11.43%
**BC specific death**	8	22.86%
**Distant relapse (any)**	9	25.71%
**Median follow up (years)**	6	

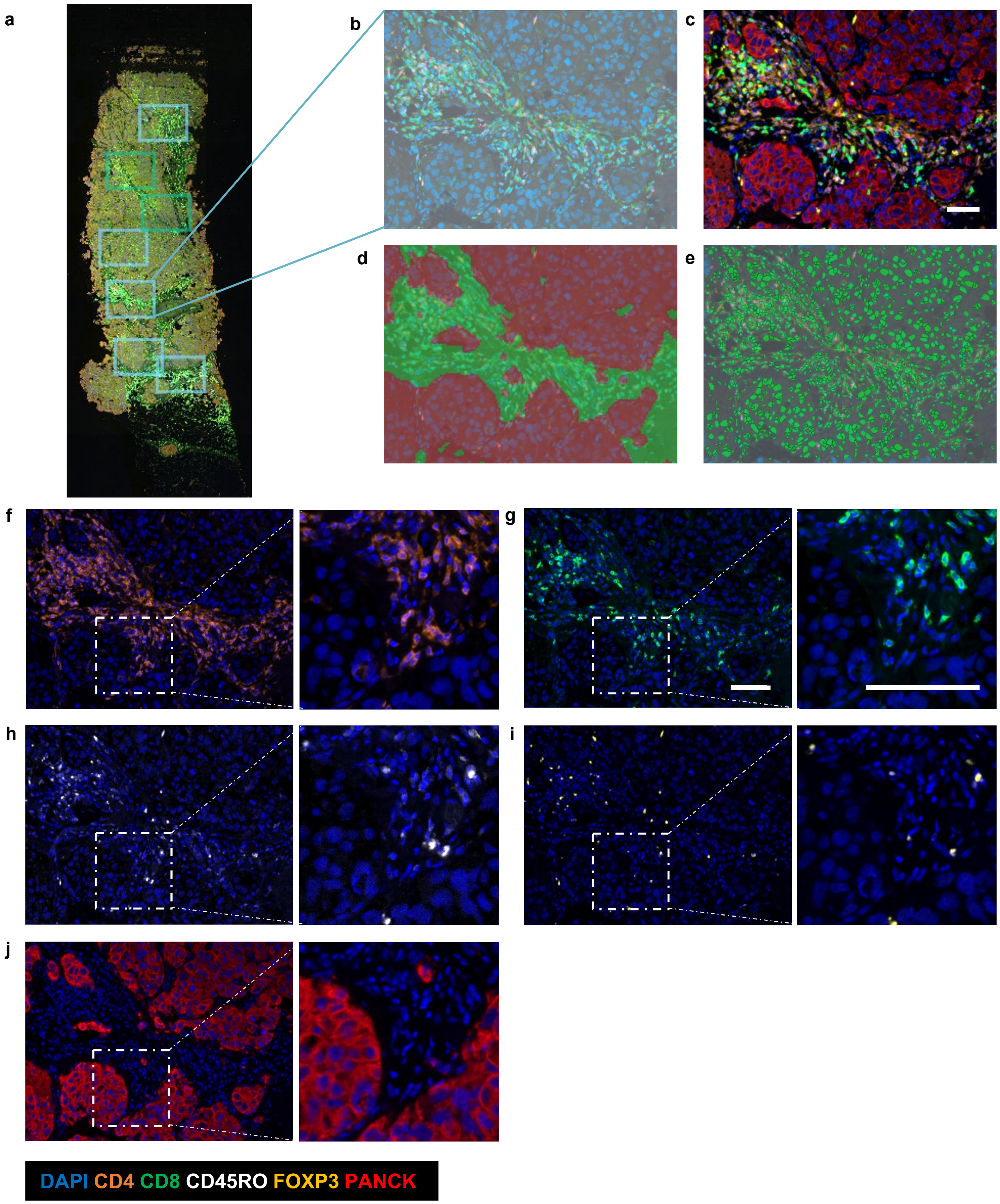

Supplementary Figure 2

Supplementary figure 2: Image acquisition and analysis using inForm software

(**a**) Whole slide scan of a 3µm section co-stained for expression of CD8, CD4, CD45RO, FOXP3, PanCK and DAPI using the OPAL system taken using the VECTRA with 4x objective. Spectrally mixed (**b**) and spectrally unmixed (**c**) image of whole slide scan section taken using the VECTRA with 20x objective. (**d**) Spectrally unmixed image segmented into tumor (red) or stroma (green) and (**e**) single cells. Single fluorescence images of CD4 (**f,** orange), CD8 (**g**, green), CD45RO (**h**, white), FOXP3 (**i**, yellow) and PanCK (**j**, red) on same region. Scale bars represent 100µm.

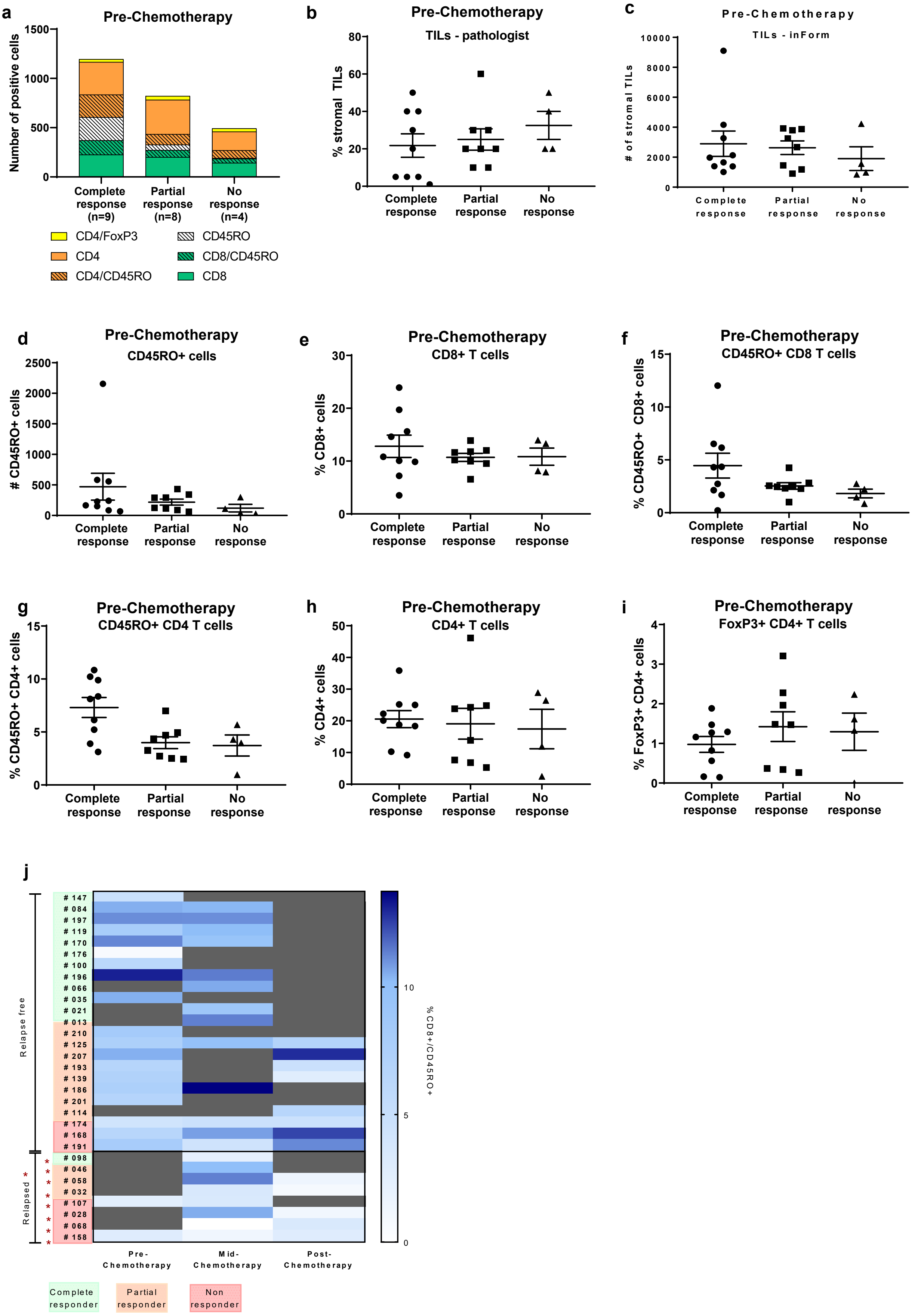

Supplementary Figure 3

Supplementary figure 3: Profiling the immune landscape throughout chemotherapy

(**a**) Bar graph of the mean number of immune populations determine by inForm software in complete, partial and non-responder TNBC primary tumors pre-chemotherapy. (**b**) Percentage of stromal TILs scored by a pathologist on a serial section H&E stained slide compared in complete, moderate and non-responder TNBC primary tumors pre-chemotherapy. (**c**) Number of stromal TILs calculated by inForm software in TNBC primary tumors pre-chemotherapy, separated based on complete, moderate or no response. (**d**) Number of CD45RO+ cells in the stroma compared in complete, moderate and non-responder TNBC primary tumors pre-chemotherapy. Percentage of CD8+ (**e**), CD45RO+CD8+ (**f**), CD45RO+CD4+ (**g**), CD4+ (**h**) and FoxP3+CD4+ (**i**) cells compared in complete, partial and non-responder TNBC primary tumors pre-chemotherapy. (**j**) Heat map representing the percentage of $CD8^{+}$ $CD45RO^{+}$ cells in the stroma of TNBC primary tumors throughout chemotherapy. Grey shading indicates no sample for evaluation.

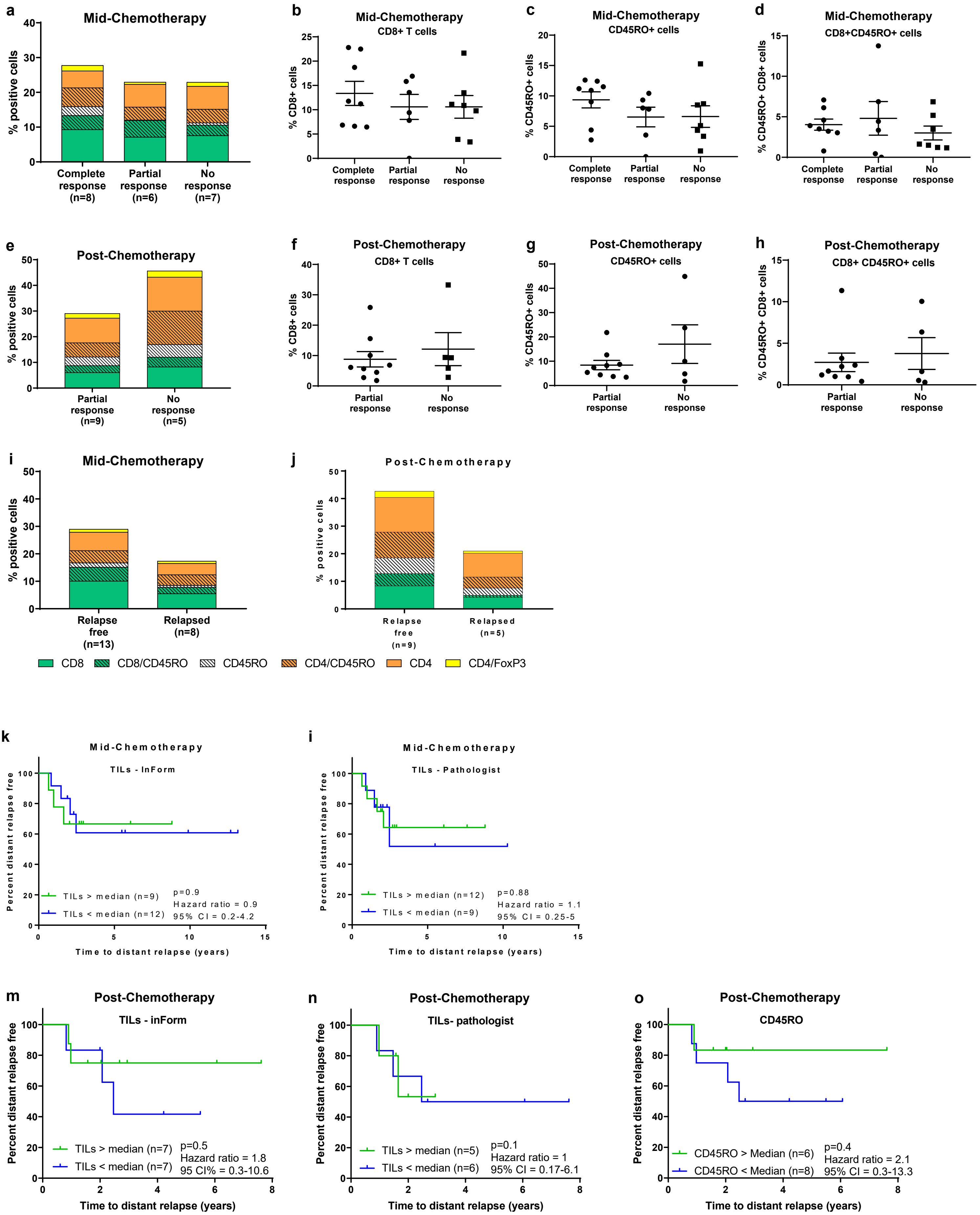

Supplementary Figure 4

Supplementary figure 4: Profiling the immune landscape throughout chemotherapy

(**a**) The mean proportion of immune populations between complete, moderate and non-responders determined by inForm software in TNBC primary tumors mid-chemotherapy. Proportion of CD8+ (**b**), CD45RO (**c**) andCD45RO+CD8+ (**d**) cells compared in complete, moderate and non-responder TNBC primary tumors mid-chemotherapy. (**e**) The mean proportion of immune populations between moderate and non-responders determined by inForm software in TNBC primary tumors post-chemotherapy. Proportion of CD8+ (**f**), CD45RO (**g**) andCD45RO+CD8+ (**h**) cells compared in moderate and non-responder TNBC primary tumors post-chemotherapy. The mean proportion of immune populations between relapse free and relapsed patients determined by inForm software in TNBC primary tumors mid-chemotherapy (**i**) and post-chemotherapy (**j**). Kaplan-Meier survival curve comparing distant relapse free survival in TNBC patients mid-chemotherapy based on proportion of TILs scored by inForm software (**k**) and by a pathologist (**l**) with groups divided by above or below the median (medians; (**k**) #1734 ; (**l**) 20%). Kaplan-Meier survival curve comparing distant relapse free survival in TNBC patients post-chemotherapy based on proportion of TILs scored by inForm software (**m**), by a pathologist (**n**) or by CD45RO (**o**) with groups divided by above or below the median (medians; (**m**) #608 ; (**n**) 20% ; (**o**) 7.8%). Error bars represent SEM.

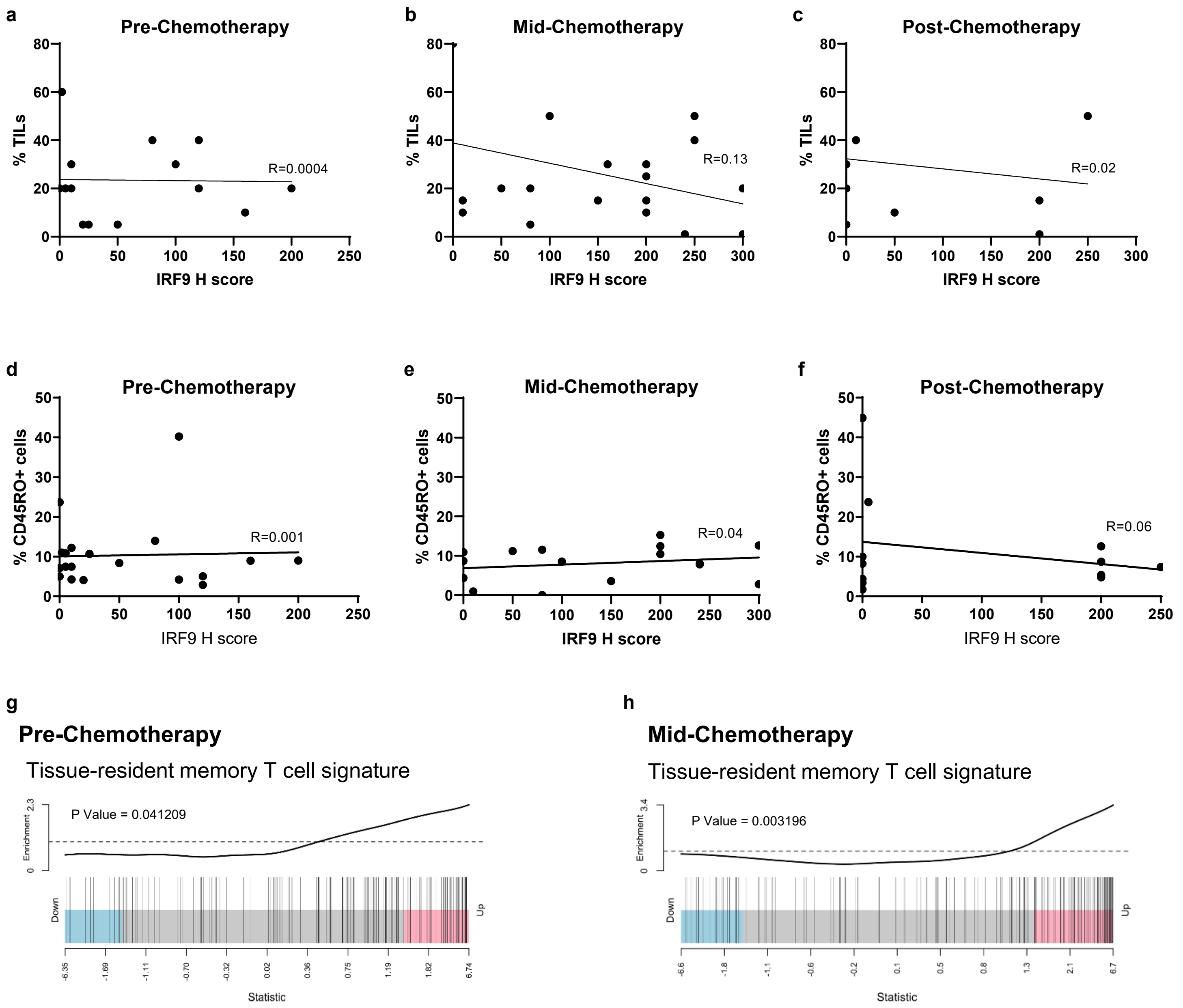

Supplementary Figure 5

Supplementary figure 5: IRF9 and chemotherapeutic response

(**a**) IRF9 H score compared between complete, partial and non-responders pre-chemotherapy. Error bars represent SEM. (**b**) Kaplan-Meier curve comparing distant relapse free survival in partial and non-responder TNBC patients who had IRF9 positive tumors or IRF9 negative tumors post chemotherapy. (**c**) IRF9 expression in complete responders post-chemotherapy was evaluated by IHC. Tissues were stained using rabbit anti-IRF9 antibody (5μg/ml), IRF9 expression visualized using DAB prior to nuclear counterstain with hematoxylin. Representative images were taken of areas that contained normal ducts, scale bars represent 50μm. (**d**) Kaplan-Meier curve comparing distant relapse free survival in all TNBC patients who had IRF9 positive tumors or IRF9 negative tumors post chemotherapy (Positive IRF9 is determined as H score > 20). p values, hazard ratios and confidence intervals calculated using a log-rank test (Mantel-Cox).

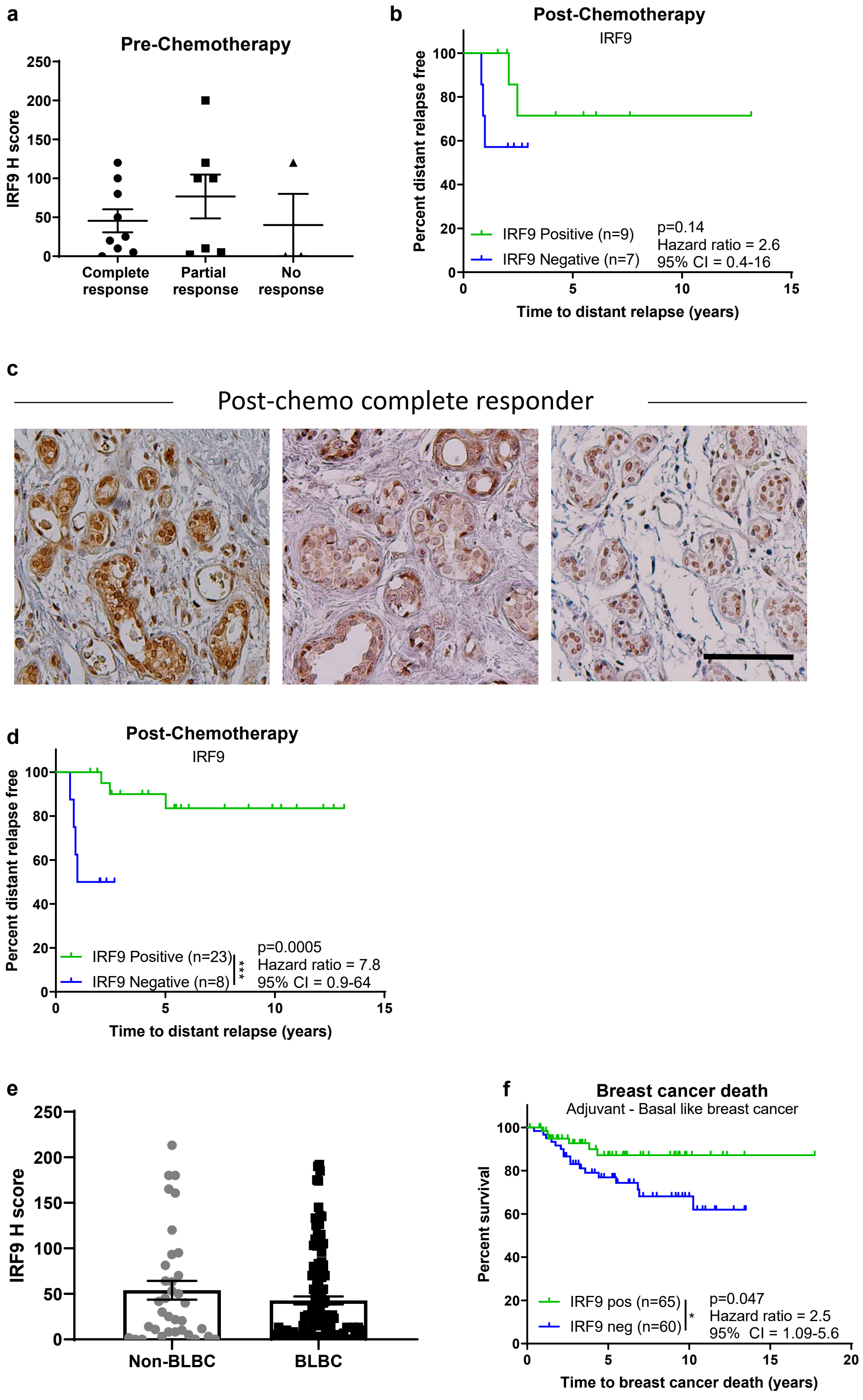

Supplementary Figure 6

Supplementary figure 6: Correlation of IRF9 with immune populations

Correlation plots of % of TILs (as scored by a pathologist) with IRF9 H score pre- (**a**), mid- (**b**) and post-chemotherapy (**c**). Correlation plots of % CD45RO+ cells (as scored by inForm) with IRF9 H score pre- (**d**), mid- (**e**) and post-chemotherapy (**f**). Line of best fit and R calculated using GraphPad. T_{RM} signature enrichment pre- (**g**) and mid-chemotherapy (**h**). Tumor samples from IRF9 positive patients (n = 5) compared with IRF9 negative patients (n = 8) (limma 'roast' gene set test). Statistic on x axis is the gene wise moderated t-statistic computed by limma, and vertical bars represent t-statistic for each gene in the gene set.

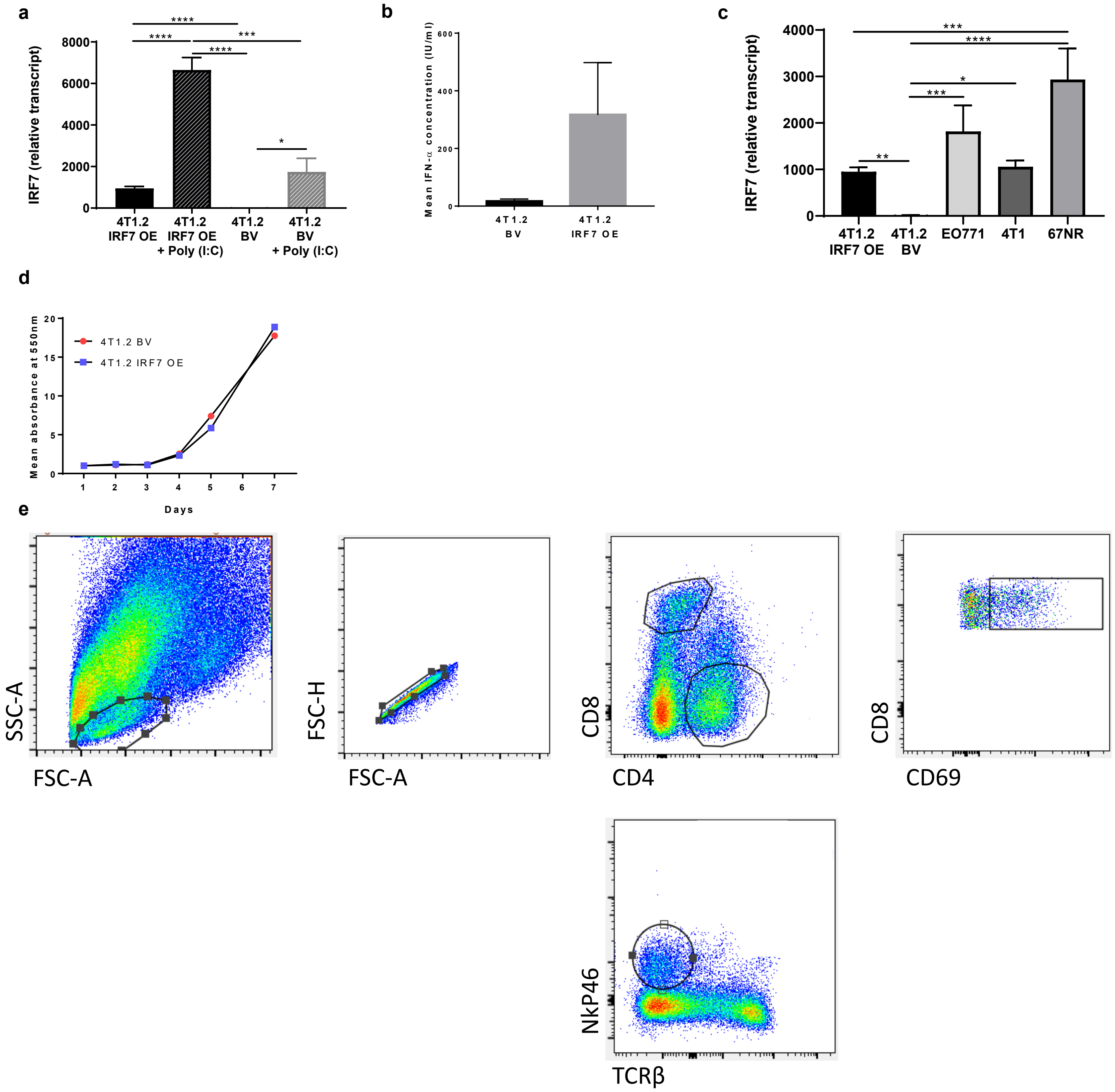

Supplementary Figure 7

Supplementary figure 7: Characterization of cells with enforced interferon signaling

(**a**) *IRF7* gene expression in 4T1.2 BV and 4T1.2 IRF7 OE cells untreated or treated with poly (I:C) (10µg/mL) relative to housekeeping gene (HPRT). (**b**) ELISA for mean secreted IFNα (IU/mL) by 4T1.2 neo BV and IRF7 OE pools. (**c**) *IRF7* gene expression in 4T1.2 BV, 4T1.2 IRF7 OE, EO771, 4T1 and 67NR cells untreated relative to housekeeping gene (HPRT). (**d**) SRB proliferation assay was conducted to demonstrate no difference in growth between the 4T1.2 BV and IRF7 OE cells. (**e)** Gating strategy to quantitate immune cells and their activation status from the primary tumor *ex vivo.*

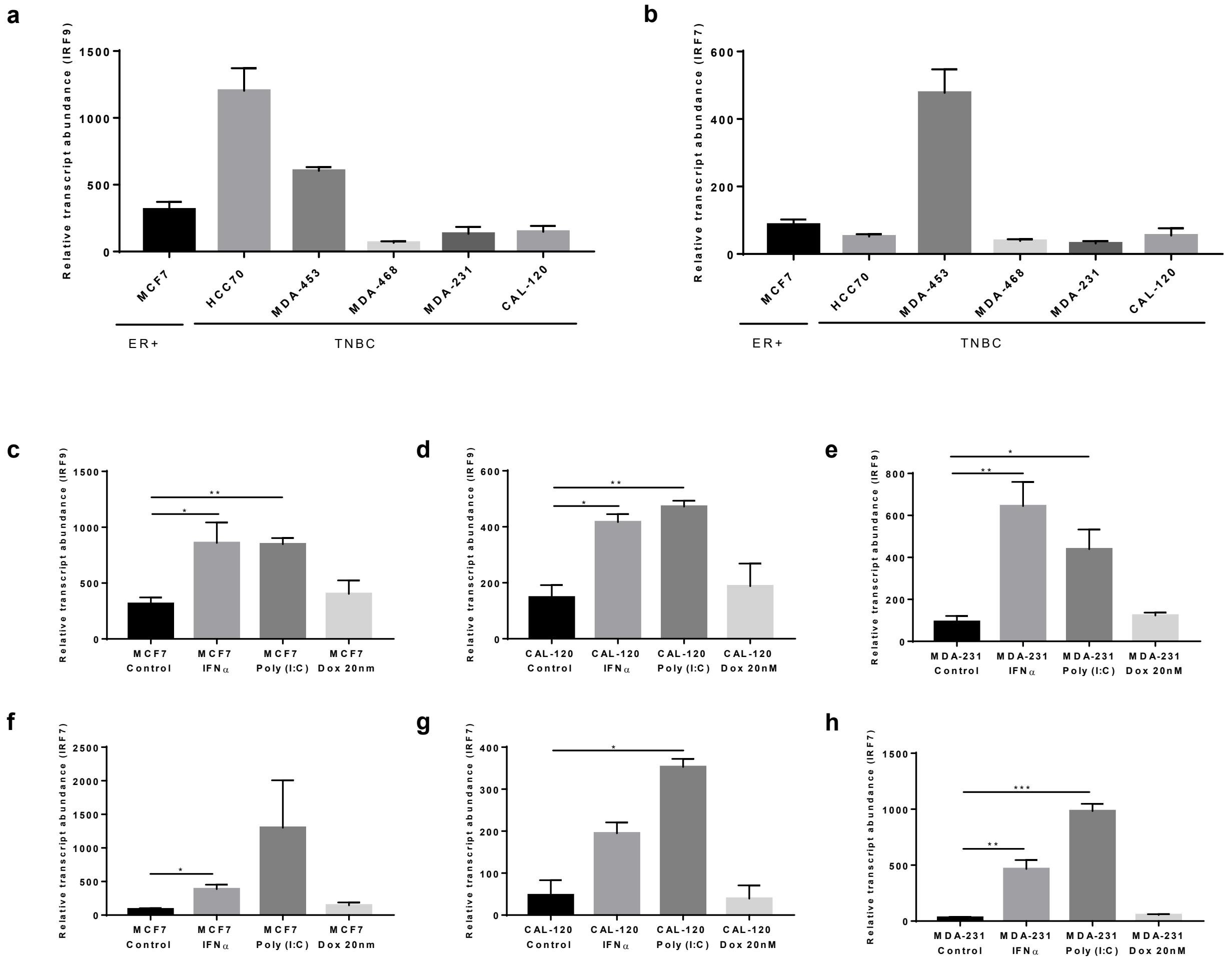

Supplementary Figure 8

Supplementary figure 8: IFN stimulation can induce IRF9 & IRF7 in human TNBC cell lines

Baseline *IRF9* (**a**) and *IRF7* (**b**) transcript in human breast cancer cell lines. *IRF9* transcript abundance in MCF7 (**c**), CAL-120 (**d**) and MDA-231 (**e**) breast cancer cell lines untreated or treated with IFNα (1000IU/ml), Poly (I:C) (10μg/ml) and doxorubicin (20nM) as indicated. *IRF7* transcript abundance in MCF7 (**f**), CAL-120 (**g**) and MDA-231 (**h**) breast cancer cell lines untreated or treated with IFNα (1000IU/ml), Poly (I:C) (10μg/ml) and doxorubicin (20nM) as indicated. Error bars represent SEM. *, $p < 0.05$; **, $p < 0.01$; ***, $p < 0.001$, using students t-test. (**i**) IRF9 H score compared between basal like breast cancer (BLBC) and non-BLBC pre-chemotherapy. Error bars represent SEM. (**j**) Kaplan-Meier curve comparing distant relapse free survival in BLBC patients who had IRF9 positive tumors or IRF9 negative tumors post chemotherapy (Positive IRF9 is determined as H score > 20). p values, hazard ratios and confidence intervals calculated using a log-rank test (Mantel-Cox).

Supplementary table 2: St Vincent's cohort patient characteristics and multivariate analysis

	St Vincent's cohort - All						St Vincent's cohort - TNBC only					
	IRF9-Negative		IRF9-Positive				IRF9-Negative		IRF9-Positive			
	Number	*Percent*	*Number*	*Percent*	*Total*	*p*	*Number*	*Percent*	*Number*	*Percent*	*Total*	*p*
	200	48.3%	214	51.7%	414		39	73.6%	14	26.4%	53	
**Age**						*0.21*						*0.28*
<45	38	19.0%	35	16.4%			4	10.3%	1	7.1%		
45-55	54	27.0%	79	36.9%			11	28.2%	8	57.1%		
55-65	59	29.5%	55	25.7%			16	41.0%	3	21.4%		
>65	48	24.0%	45	21.0%			8	20.5%	2	14.3%		
**Grade**						***0.0001***						*0.43*
1	26	13.0%	36	16.8%			1	2.6%	1	7.1%		
2	54	27.0%	96	44.9%			1	2.6%	8	57.1%		
3	109	54.5%	69	32.2%			36	92.3%	5	35.7%		
Unknown	10	5.0%	13	6.1%			1	2.6%	0	0.0%		
**Tumour size**						***0.0003***						*0.3*
T1	76	38.0%	123	57.5%			11	28.2%	7	50.0%		
T2	107	53.5%	75	35.0%			20	51.3%	6	42.9%		
T3	13	6.5%	11	5.1%			7	17.9%	1	7.1%		
Unknown	4	2.0%	5	2.3%			1	2.6%	0	0.0%		
**LN involvement**						***0.038***						*0.24*
Negative	84	42.0%	107	50.0%			19	48.7%	10	71.4%		
Positive	111	55.5%	93	43.5%			19	48.7%	4	28.6%		
Unknown	5	2.5%	14	6.5%			1	2.6%	0	0.0%		
**ER status**						***0.041***						*NA*
Negative	67	33.5%	28	13.1%			39	100.0%	14	100.0%		
Positive	116	58.0%	170	79.4%			0	0.0%	0	0.0%		
**PR status**						***0.017***						*NA*
Negative	74	37.0%	58	27.1%			39	100.0%	14	100.0%		
Positive	105	52.5%	139	65.0%			0	0.0%	0	0.0%		
**HER2 status**						*0.09*						*NA*
Negative	154	77.0%	177	82.7%			39	100.0%	14	100.0%		
Positive	42	21.0%	31	14.5%			0	0.0%	0	0.0%		
**BC specific death**	58	29.0%	48	22.4%		*0.08*	16	41.0%	2	14.3%		*0.07*
**Local relapse breast**	12	6.0%	16	7.5%		*0.12*	7	17.9%	0	0.0%		*0.31*
**Distant relapse (any)**	46	23.0%	36	16.8%		*0.08*	18	46.2%	1	7.1%		***0.04***

	BC-Specific Survival			Metastasis-free Survival			Relapse-free Survival		
**Clinicopathological variable**	*Hazard Ratio*	*95% CI*	*p-value*	*Hazard Ratio*	*95% CI*	*p-value*	*Hazard Ratio*	*95% CI*	*p-value*
IRF9 (neg vs pos)	26.8	1.63 - 439.02	**0.021**	7.5	0.28 - 204.8	0.078	n.c.	n.c.	0.983
Lymph node status (pos vs neg)	4.6	1.22 - 17.58	**0.024**	1.8	0.62 - 5.04	0.317	0.7	0.10 - 4.57	0.692
Histological grade (3 vs 1/2)	0.1	0.01 - 0.96	**0.047**	0.5	0.02 - 13.29	0.221	n.c.	n.c.	0.991
Tumour size (<20mm vs ≥20mm)	0.8	0.27 - 2.64	0.774	1.9	0.58 - 6.20	0.236	1.2	0.17 - 7.73	0.881
Age (<55y vs ≥55y)	0.6	0.23 - 1.76	0.385	1.4	0.51 - 3.65	0.548	3.9	0.43 - 35.15	0.227

n.c. = not calculable

Supplementary table 3: RPAH cohort patient characteristics

	RPAH TNBC Cohort					
	IRF9-Negative		IRF9-Positive			
	Number	*Percent*	*Number*	*Percent*	*Total*	*p*
	76	47.8%	83	52.2%	159	
**Age**						0.23
<45	13	17.1%	18	21.7%		
45-55	27	35.5%	28	33.7%		
55-65	13	17.1%	11	13.3%		
>65	22	28.9%	11	13.3%		
**Grade**						0.84
1	1	1.3%	1	1.2%		
2	6	7.9%	9	10.8%		
3	67	88.2%	73	88.0%		
Unknown	2	2.6%	0	0.0%		
**Tumour size**						0.98
T1	24	31.6%	26	31.3%		
T2	45	59.2%	51	61.4%		
T3	6	7.9%	6	7.2%		
Unknown	1	1.3%	0	0.0%		
**LN involvement**						0.75
Negative	39	51.3%	47	56.6%		
Positive	33	43.4%	31	37.3%		
Unknown	4	5.3%	5	6.0%		
**BC specific death**	21	27.6%	9	10.8%		**0.04**
**Local relapse breast**	10	13.2%	3	3.6%		**0.06**
**Distant relapse (any)**	14	18.4%	10	12.0%		0.5

References

1. D. C. Rio, M. Ares, G. J. Hannon, T. W. Nilsen, Purification of RNA Using TRIzol (TRI Reagent), *Cold Spring Harb. Protoc.* **2010**;2010:pdb.prot5439.

2. V. Vichai, K. Kirtikara, Sulforhodamine B colorimetric assay for cytotoxicity screening., *Nat. Protoc.* **2006**;1:1112–1116.
